# Hepatitis B virus infection disrupts homologous recombination in hepatocellular carcinoma by stabilizing resection inhibitor ADRM1

**DOI:** 10.1172/JCI171533

**Published:** 2023-12-01

**Authors:** Ming Zeng, Zizhi Tang, Laifeng Ren, Haibin Wang, Xiaojun Wang, Wenyuan Zhu, Xiaobing Mao, Zeyang Li, Xianming Mo, Jun Chen, Junhong Han, Daochun Kong, Jianguo Ji, Antony M. Carr, Cong Liu

**Affiliations:** 1 Department of Pediatrics, Key Laboratory of Birth Defects and Related Diseases of Women and Children (Ministry of Education), West China Second University Hospital, Sichuan University, Chengdu, China.; 2 Department of Immunology, Shanxi Hospital Affiliated to Cancer Hospital, Chinese Academy of Medical Sciences, Taiyuan, China.; 3 Department of Pediatric Surgery, Wuhan Children’s Hospital, Huazhong University of Science and Technology, Wuhan, China.; 4 State Key Laboratory of Protein and Plant Gene Research, School of Life Sciences, Peking University, Beijing, China.; 5 State Key Laboratory of Biotherapy, West China Hospital, Sichuan University, Chengdu, China.; 6 MOE Laboratory of Biosystems Homeostasis & Protection and Innovation Center for Cell Signaling Network, College of Life Sciences, Zhejiang University, Hangzhou, China.; 7 Genome Damage and Stability Centre, School of Life Sciences, University of Sussex, Brighton, United Kingdom.

**Keywords:** Hepatology, Virology, DNA repair, Liver cancer, Ubiquitin-proteosome system

## Abstract

Many cancers harbor homologous recombination defects (HRDs). A HRD is a therapeutic target that is being successfully utilized in treatment of breast/ovarian cancer via synthetic lethality. However, canonical HRD caused by BRCAness mutations do not prevail in liver cancer. Here we report a subtype of HRD caused by the perturbation of a proteasome variant (CDW19S) in hepatitis B virus–bearing (HBV-bearing) cells. This amalgamate protein complex contained the 19S proteasome decorated with CRL4^WDR70^ ubiquitin ligase, and assembled at broken chromatin in a PSMD4^Rpn10^- and ATM-MDC1-RNF8–dependent manner. CDW19S promoted DNA end processing via segregated modules that promote nuclease activities of MRE11 and EXO1. Contrarily, a proteasomal component, ADRM1^Rpn13^, inhibited resection and was removed by CRL4^WDR70^-catalyzed ubiquitination upon commitment of extensive resection. HBx interfered with ADRM1^Rpn13^ degradation, leading to the imposition of ADRM1^Rpn13^-dependent resection barrier and consequent viral HRD subtype distinguishable from that caused by *BRCA1* defect. Finally, we demonstrated that viral HRD in HBV-associated hepatocellular carcinoma can be exploited to restrict tumor progression. Our work clarifies the underlying mechanism of a virus-induced HRD subtype.

## Graphical abstract

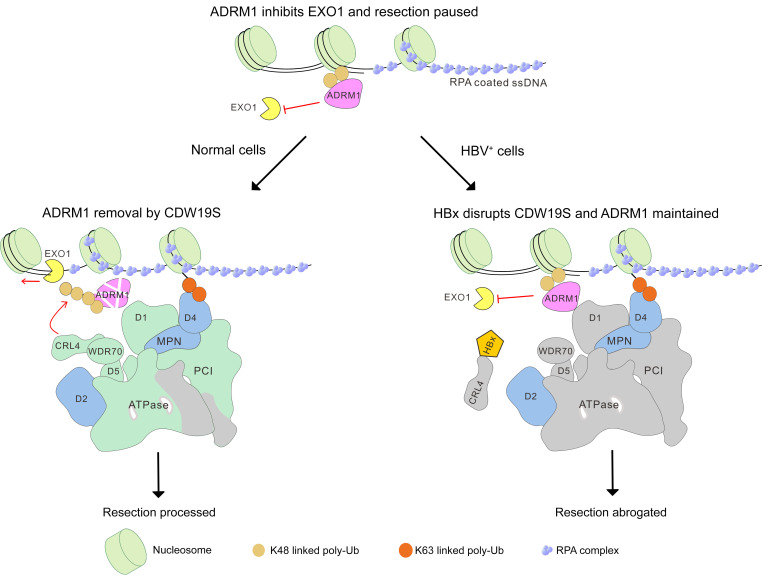

## Introduction

Long-term liver infection with hepatitis B virus (HBV) predisposes carriers to hepatocellular carcinoma (1). Therapy for late-stage HBV-associated hepatocellular carcinoma remains a major challenge, with poor therapy responses and low overall survival (2). HBV subverts the Cullin4-DDB1-RING (CRL4) ubiquitin ligase for viral purposes via the oncoprotein HBx (3). Among the CRL4 subcomplexes that subsequently show reduced function is CRL4^WDR70^, a regulator for homologous recombination and chromatin remodeling (4). In HBV^+^ cells, enrichment of homologous recombination (HR) factors including RPA, RAD51, and EXO1 at DNA double-strand breaks (DSBs) is compromised, correlated to reduced CRL4^WDR70^ activity and DNA end resection (5). However, the mechanistic role of CRL4^WDR70^ in DNA repair remains undetermined.

Misregulation of DSB repair compromises chromosomal stability (6) and is often characterized by altered usage of non-homologous end joining (NHEJ) and HR (7). DNA end processing by 5′-to-3′ resection governs HR commitment by generating RPA-coated single-stranded DNA (ssDNA) that subsequently loads RAD51 to form a filament that enables homology search (8). The cell cycle position and the chromatin context surrounding the DSB site influence ssDNA production (9, 10). DSBs occurring in G_1_ are repaired by NHEJ, while those occurring after replication are repaired by NHEJ and/or HR. The choice of pathway is regulated by the competitive occupancy of 53BP1 and BRCA1 at DNA breaks (11–13). A new therapeutic strategy, synthetic lethality (SL), has recently been introduced for cancer subtype–specific chemotherapy, and this was first exemplified by the treatment of homologous recombination defect (HRD) breast cancers carrying *BRCA1/2* mutations (BRCAness) with PARP inhibitors (PARPis), including olaparib and talazoparib (14).

DNA repair is also regulated by the 19S regulatory particle (RP), a constituent of the 26S proteasome that degrades ubiquitin-tagged proteins. Distinct from the protease activity sequestered in 20S core particle (CP), the canonical 19S RP recognizes ubiquitinated targets and deubiquitinates and positions them for translocation and unfolding to allow degradation by the CP (15). The 19S RP is subdivided into a “base” that is constituted of 6 paralogous AAA^+^ ATPases (PSMC1–6) plus several non-ATPase proteins (PSMD1^Rpn2^, PSMD2^Rpn1^, and ADRM1^Rpn13^) and a “lid” containing PCI domain proteins (PSMD3, 6, 8, 11–13), MPN domain proteins (PSMD7^Rpn8^ and POH1^Rpn11^), plus the non-PCI/MPN domain subunits including DSS1 (also known as Rpn15 or Sem1) (16, 17). The base and lid are conformationally dynamic and together bind a further subunit, the ubiquitin receptor PSMD4^Rpn10^.

As introduced above, in addition to regulating proteolysis, the RP also performs non-proteolytic roles in the context of chromatin. This was originally identified from the recruitment of a subset of RP proteins to the GAL1-10 promoter, implying a direct role in RNA polymerase II transcription (18). In the context of DNA repair, the 19S has subsequently been shown to modulate the efficiency of both DSB and nucleotide excision repair (19–21). DSS1^Sem1^ and POH1^Rpn11^ locate at DSB sites and regulate repair activities of RAD52/Pol4 and 53BP1/RAP80, respectively (22–25). The number and diversity of 19S-associating proteins and functions have obscured the elucidation of its mechanism in chromatin biology, and a comprehensive model depicting its interplay with DNA repair machinery is lacking.

Here we provide evidence that CRL4^WDR70^ forms a specific complex with the break-associated 19S proteasome (subsequently referred to as CDW19S; CULLIN4A-DDB1-WDR70-19S) that favors HR via end processing. 19S RP controls both MRE11 and EXO1 nucleases, and CRL4^WDR70^ engages with an EXO1-specific module of RPs to catalyze the ubiquitination and degradation of ADMR1^Rpn13^, a 19S-associated ubiquitin receptor that we identify as a resection barrier factor. We show that HBx disintegrates CUL4A-DDB1 from CDW19S and leaves scaffold-free WDR70-19S on damaged DNA. As such, HBx retards the clearance of ADRM1^Rpn13^ from DNA breaks, whereby a special HRD subtype is produced distinct from that caused by *BRCA1* deficiency. Similar to the addiction of BRCAness cells to PARP functions, the viral HRD sensitizes HBV^+^ cells to PARPi. These data uncover a viral HRD subtype resulting from failed clearance of resection inhibitors.

## Results

### HBx induces HRD by perturbing the balanced choice of DSB repair.

**Figure 1:**
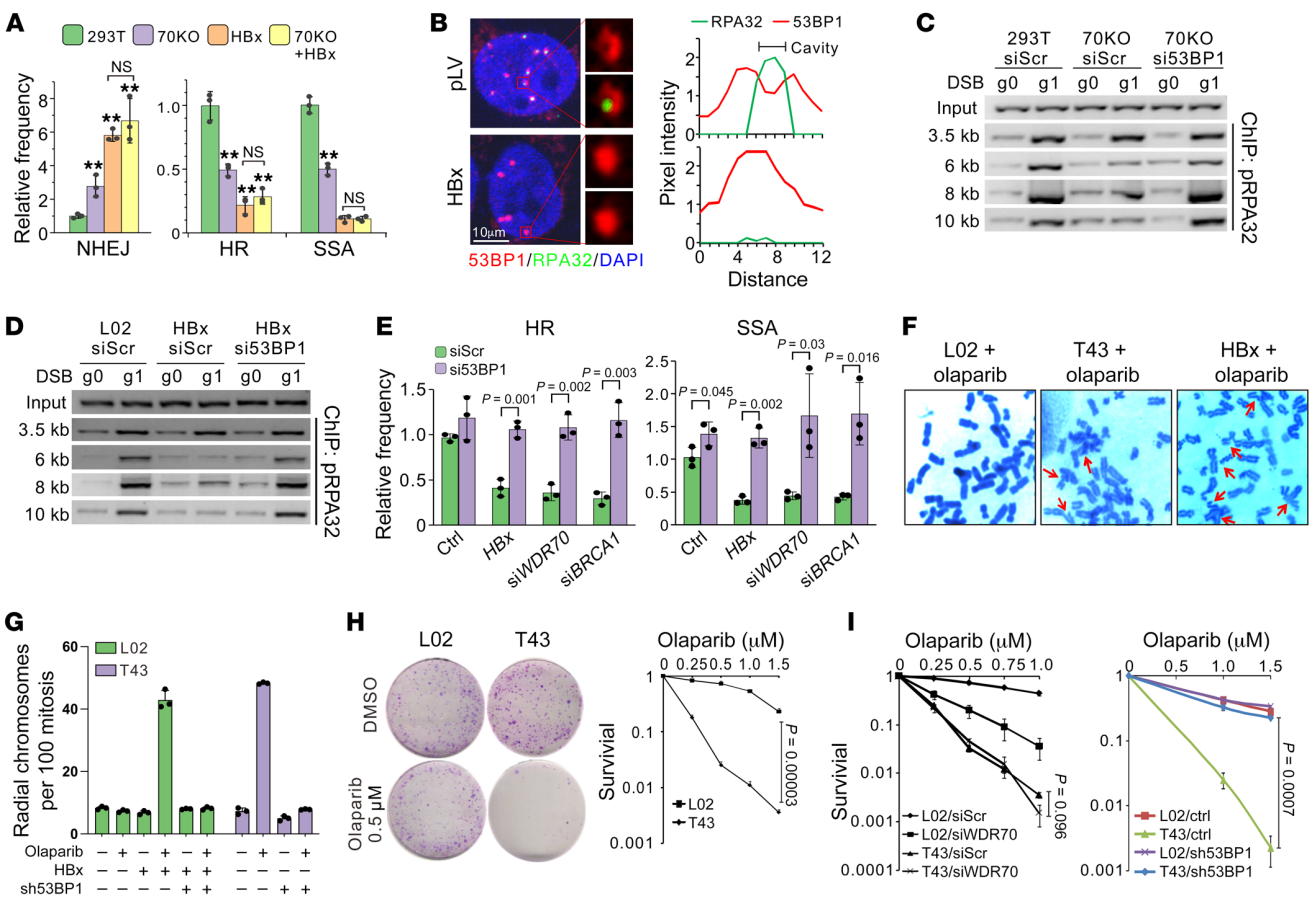
Interference with CRL4^WDR70^ by HBx induces a viral HRD. (**A**) Repair frequency of indicated pathways in *WDR70*-knockout or *HBx*-expressing cells relative to control cells (293T). **P <0.05 by 2-tailed *t* test. NS, no statistic significance. (**B**) Left: Example confocal images showing 53BP1 (red) and RPA32 (green) IRIF in *HBx-*expressing L02 cells 8 hours after IR. Soluble nuclear proteins were preextracted with 0.1% Triton X-100. Scale bar: 10 μm. Right: Pixel intensity (vertical) across the maximal central line of individual IRIFs. Precipitation of red line (53BP1) and rising of green line (RPA32) along the vertical axis indicate the central cavity. (**C** and **D**) ChIP assay depicting p-RPA32 chromatin loading at indicated distance from the DSB upon expression of gRNA (g1) targeting the *PPP1R12C*/*p84* locus. *WDR70-*knockout or control 293T cells (**C**) and L02 cells expressing *HBx* (**D**) were cotransfected with si*53BP1* or control siRNA (siScr). (**E**) Relative HR/SSA efficiency for L02 cells pretreated with *HBx*, si*WDR70*, or si*BRCA1* and concomitant silencing of *53BP1*. (**F** and **G**) Representative images (**F**) and quantification (**G**) of aberrant chromosomes in the indicated cells cotreated with olaparib (1 μM) and/or sh*53BP1*. (**H**) Giemsa staining for colony formation (left) and survival curves (right) of control L02 (HBV^–^) and T43 (HBV^+^) cells subjected to olaparib treatment. Survival at endpoints was analyzed for statistical significance. *n* = 3 biological repeats; error bars indicate SD; *t* test. (**I**) Survival curves for L02 and T43 cells treated with si*WDR70* (left) or sh*53BP1* (right) with simultaneous exposure to indicated concentrations of olaparib. *n* = 3 experimental repeats; error bars indicate SD; *P* values by *t* test are shown for indicated groups.

To assess the impacts of HBV/HBx on DSB repair, an I-*Sce*I–induced DSB system was used to measure sister chromatid repair (HR), single-strand annealing (SSA), and NHEJ efficiencies (26). T43 hepatocytes, which harbor integrated HBV genomes (5), displayed a modest decrease in HR and SSA relative to the parental HBV-free L02 cells (Supplemental Figure 1A; supplemental material available online with this article; https://doi.org/10.1172/JCI171533DS1). To establish whether this HBV-induced HR deficiency can be attributed, at least in part, to HBx ablating CRL4^WDR70^, we expressed *HBx* in HEK293T cells and in *WDR70*-knockout derivatives (Figure 1A). *HBx* biased repair away from HR/SSA and toward NHEJ as expected. *WDR70* deletion did not affect the repair profile beyond that seen when *HBx* is expressed in 293T cells, consistent with an epistatic relationship.

53BP1 establishes a resection barrier at DSB ends after ionizing radiation (IR). To activate HR, 53BP1 is subsequently displaced from the focal center of ionizing radiation–induced foci (IRIF) in a BRCA1-dependent manner (23). This 53BP1 loss correlates with the accumulation of ssDNA binding factor (RPA32) within the central cavity of the focus, a process that is dependent on functional resection. Upon HBV infection, *HBx* expression, or *WDR70* loss, a reduction in the size of central cavities of 53BP1 IRIF that is positive for RPA32 was observed, indicative of impaired displacement of 53BP1 by pro-HR factors (Figure 1B and Supplemental Figure 1B). Consistent with the antagonistic role of BRCA1 against 53BP1 (11), *HBx* expression or direct compromising of CRL4^WDR70^ diminished attraction of BRCA1 to DSBs (Supplemental Figure 1C).

HR defects caused by *BRCA1* mutations can be rescued by removal of the *53BP1*-mediated resection barrier (27). Similar to this situation, in *HBx-*expressing cells, depleting *53BP1* restored camptothecin-induced (CPT-induced) RPA32 phosphorylation (p-RPA32) respective to controls (Supplemental Figure 1D), and similarly restored IRIF of p-RPA32 and RAD51 recombinase in cells transfected with small interfering RNA of WDR70 (si*WDR70*) (Supplemental Figure 1E). We evaluated DSB loading of 53BP1 in *WDR70*-knockout and *HBx-*expressing cells using chromatin immunoprecipitation (ChIP) assay developed for CRISPR-induced DSBs at the PPP1R12C/p84 locus (5), where enhanced 53BP1 binding was detected at 0.5–10 kb from the break in comparison with their respective controls (Supplemental Figure 1F). When *53BP1* was concomitantly knocked down in *WDR70*-knockout and *HBx-*expressing cells, restricted p-RPA32 association within 6–10 kb from the break site was reactivated to levels comparable to those in wild-type cells (Figure 1, C and D). Resembling the revitalization of BRCAness HRD by *53BP1* depletion, simultaneous silencing of *53BP1* restored HR/SSA efficacies in *HBx* expression and si*WDR70* cells (Figure 1E). We conclude that HBV/HBx-induced viral HRD shares the common defect with BRCAness HRD in terms of 53BP1 accumulation.

In the context of *BRCA1* ablation, preferential channeling of DSB repair to error-prone NHEJ promotes the generation of toxic chromosomal structures that are exacerbated upon PARP inhibition (27, 28). Similarly, chromosomal aberrations were synergistically exacerbated in *HBx*-expressing L02 and T43 cells upon PARPi (olaparib) addition, and this was suppressible by *53BP1* ablation (Figure 1, F and G). Strikingly, T43 cells were hypersensitive to a range of PARPis when compared with HBV-free L02 cells (Figure 1H and Supplemental Figure 1G), revealing a strong synthetic lethality between HBV/HBx and PARP inhibition. A second HBV^+^ cell line (HepG2.2.15) displayed comparable SL phenotype relative to its parental line (HepG2; Supplemental Figure 1H). This SL subtype was linked to CRL4^WDR70^, as si*WDR70* sensitized L02 cells to PARPi but showed no additional effect on T43 cell PARPi sensitivity (Figure 1I, left). T43 sensitivity to PARPi was rescued by sh*53BP1* (Figure 1I, right), reinforcing the contribution of DSB-associated 53BP1 in viral HRD induction.

### Assembling CDW19S on break-associated chromatin.

**Figure 2:**
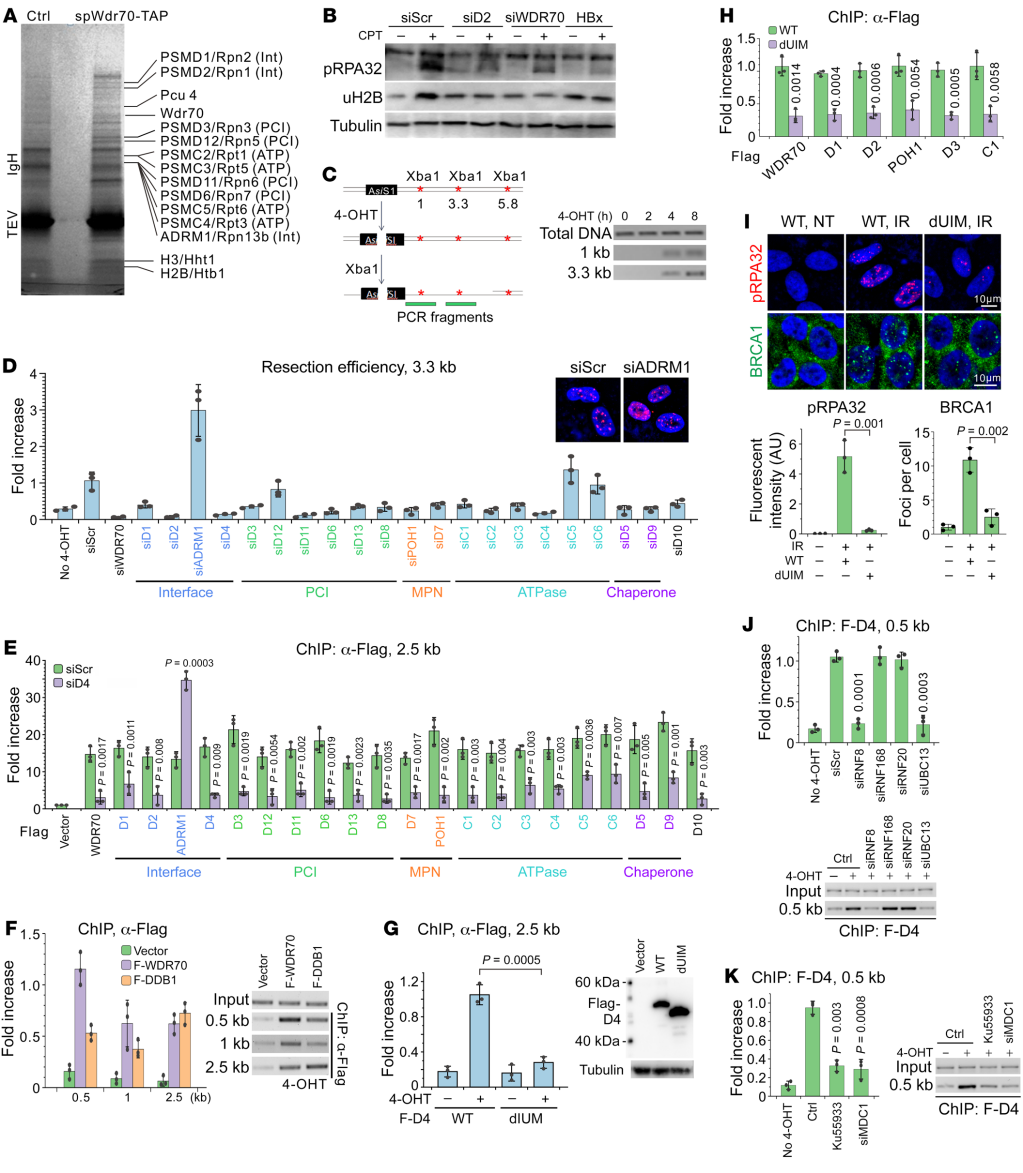
CDW19S engages DSB-proximal chromatin. (**A**) TAP-affinity-purified spWdr70-interacting proteins separated by gradient SDS-PAGE. Proteins identified by MALDI-TOF mass spectrometry are shown on the right. See peptide coverage in Supplemental Table 1. Subdomains of interface (Int), PCI, MPN, and ATPase (ATP) are indicated for RP subunits using human and yeast nomenclatures. IgH, heavy chain of rabbit IgG; TEV, tobacco etch virus endopeptidase. (**B**) Immunoblotting for p-RPA32 and H2B monoubiquitination (uH2B) in L02 cells with indicated siRNA and CPT treatment. (**C**) Left: Illustration for *Xba*I-based resection assay at the selected *Asi*SI-dependent DSB site (chromosome 1: 89,458,595–89,458,603). Right: Example of monitoring of ssDNA production by semiquantitative PCR. (**D**) Quantification for *Xba*I-based resection assay showing DSB processing in DIvA cells depleted for the indicated CDW19S subunits. Inset: Excessive p-RPA32 immunostaining implies hypersection in si*ADRM1^Rpn13^* RPE1 cells. Data normalized to control (siScr) with 4-OHT induction. Original magnification (inset): ×400. (**E**) ChIP assay 2.5 kb distal to an *Asi*SI-induced DSB showing break association of FLAG-tagged CDW19S subunits upon *PSMD4^Rpn10^* ablation relative to control transfection. DIvA cells all treated with 4-OHT. (**F**) Left: ChIP assay for FLAG-tagged WDR70/DDB1 at indicated distances from *Asi*SI-induced DSB ends. Right: Representative PCR products. (**G**) ChIP assay of FLAG-PSMD4^Rpn10^ 2.5 kb from an *Asi*SI-induced DSB upon *PSMD4^WT^* or *PSMD4^dUIM^* expression. Anti-FLAG immunoblotting is shown in the right panel. (**H**) Equivalent ChIP assay for indicated CDW19S subunits in the presence of *PSMD4^WT^* or *PSMD4^dUIM^* expression. (**I**) Top: Representative images of p-RPA32 and BRCA1 IRIF in the presence of *PSMD4^Rpn10^* or *PSMD4^dUIM^* (4 hours after IR). Nuclei counterstained with DAPI. Scale bars: 10 μm. Bottom: Quantification of fluorescent intensity or foci numbers. In **H** and **I**, *PSMD4* plasmids are FLAG-less and siRNA resistant, and cells were cotransfected with si*PSMD4^Rpn10^*. (**J** and **K**) PSMD4^Rpn10^ enrichment upon 4-OHT induction at 0.5 kb from an *Asi*SI-induced DSB after treatment with the indicated siRNA or inhibitors.

To establish how viral HRD is induced and CRL4^WDR70^ interplays with the repair machinery, we purified proteins associated with TAP-tagged Wdr70 from fission yeast, given the evolutionary conservation of CRL4^WDR70^ (4). *Ddb1*, the adaptor that links Wdr70 and Pcu4 (Cullin4 homolog), was deleted to disable the ubiquitin ligase activity, thus preventing the degradation of binding factors. MALDI-TOF mass spectrometry of sliced gel bands identified 3 categories of proteins (Figure 2A and Supplemental Table 1): histone species (Htb1^H2B^, Hht1^H3.1^), ubiquitination factors (note we saw low coverage for Pcu4 and abundant ubiquitin), and, unexpectedly, a range of proteins derived from 19S RP of the proteasome. The panel of RP subunits encompasses the main RP subcomplexes including PCI, MPN, and ATPase domain proteins. Notably, no peptides from the proteolytic 20S core particle were retrieved.

Coimmunoprecipitation for WDR70/DDB1 in cells ectopically expressing FLAG-tagged 19S subunits solidified the conservation of CRL4^WDR70^-19S interaction in human cells (Supplemental Figure 2A). Consistent with a role in DSB repair, these interactions were enhanced upon CPT treatment (Supplemental Figure 2B). A functional importance of the interaction is implied by the observation that, as shown previously for *HBx* expression and si*WDR70* (5), depleting the proteasomal *PSMD2^Rpn1^* subunit inhibited pro-HR events including RPA32 phosphorylation and H2B monoubiquitylation (Figure 2B). We thus conclude that CRL4^WDR70^ and the 19S particle form a complex (CDW19S) that influences DSB repair.

To survey the function of the CDW19S complex at DSBs, the DIvA system, where DNA breaks are generated by ER-tagged *Asi*SI endonuclease upon its nuclear import following 4-OHT treatment (29), was used to analyze a specific *Asi*SI site on chromosome 1 (89,458,595–89,458,603, reference genome hg19) by ChIP. Four hours after 4-OHT treatment, p-RPA32 was observed in control cells between 0.5 and 5 kb from the break, whereas loss of *WDR70* affected distal (2.5–5 kb) but not proximal (0.5–1 kb) deposition (Supplemental Figure 2C). Distal resection (3.3 kb) was further analyzed by digestion of genomic DNA with *Xba*I, which is inactive on ssDNA (Figure 2C). Uncut ssDNA was quantified by PCR across the digestion site, and 4 hours after induction, control cells showed evidence of ssDNA at 1 kb and 3.3 kb from the break site. In this resection system, knockdown of the majority of CDW19S subunit genes, plus those encoding the associated chaperones (PSMD5^Hsm3^ and PSMD9^Nas2^) and PSMD10 proteins, exhibited si*WDR70*-like resection defects relative to si*Scramble* (Figure 2D). *ADRM1^Rpn13^* depletion stood out, displaying hyperactive ssDNA production relative to control cells. No function for PSMD12^Rpn5^, PSMC5^Rpt6^, and PSMC6^Rpt4^ was observed, despite significant mRNA ablation (Supplemental Figure 2D). Moreover, ChIP assay revealed that all RP and WDR70/DDB1 proteins (FLAG-tagged) were enriched at 2.5 kb distal to DSBs following *Asi*SI nuclear import (Figure 2, E and F). We conclude that CRL4^WDR70^ decorates 19S RP in a stable CDW19S complex to stimulate resection.

### PSMD4^Rpn10^ recruits CDW19S in an ATM-MDC1-RNF8–dependent manner.

19S RP is known to be targeted to chromatin and interplay with DSB-associated ubiquitin conjugates (24, 30). We speculated that the ubiquitin binding directs the RP to the DSBs. PSMD4^Rpn10^, an integral ubiquitin receptor in the RP lid that recruits K63- or K48-linked ubiquitin targets to the proteasome, is a promising candidate (31). Knockdown of *PSMD4^Rpn10^* abolished DSB recruitment of WDR70 and all RP subunits except ADRM1^Rpn13^ (Figure 2E). In contrast, PSMD4^Rpn10^ was recruited to DSBs irrespective of siRNA treatment for any of the CDW19S subunits tested (Supplemental Figure 2E). PSMD4^Rpn10^ recognizes ubiquitin chains via a ubiquitin-interacting motif (UIM), mutation of which abrogates ubiquitin affinity (32). Ectopically expressed UIM-deletion mutant (*PSMD4^dUIM^*) dissociated from DSBs (Figure 2G) and suppressed DSB assembly of CDW19S complex (Figure 2H). Consistent with this, PSMD4^dUIM^ expression phenocopied si*WDR70*, repressing resection as monitored by p-RPA32 and BRCA1 IRIF (Figure 2I).

To establish whether damage signaling was prerequisite for CDW19S DSB association, we evaluated the abundance of PSMD4^Rpn10^ at break sites when early damage signaling was interfered with. Silencing the damage-responsive E3 enzyme (RNF8) and its partner E2 (UBC13), rather than RNF168 or RNF20, effectively abolished PSMD4^Rpn10^ loading 0.5 kb from the DSB (Figure 2J). RNF8-UBC13 catalyzes K63-polyubiqutination chains, suggesting that PSMD4^Rpn10^ is attracted to DSB-associated K63-modified proteins. The RNF8 FHA domain docks to TQxF clusters on MDC1 following their ATM-dependent phosphorylation (33). Consistent with this, both ATM kinase activity (kinase inhibitor KU55933) and MDC1 (si*MDC1*) were required for the DSB attraction of PSMD4^Rpn10^ (Figure 2K). We conclude that the assembly of CDW19S is initiated at break proximity by PSMD4^Rpn10^ recognition of K63 species that is ATM-MDC1-RNF8 dependent.

### CDW19S is functionally segregated into MRE11- and EXO1-regulatory modules.

**Figure 3:**
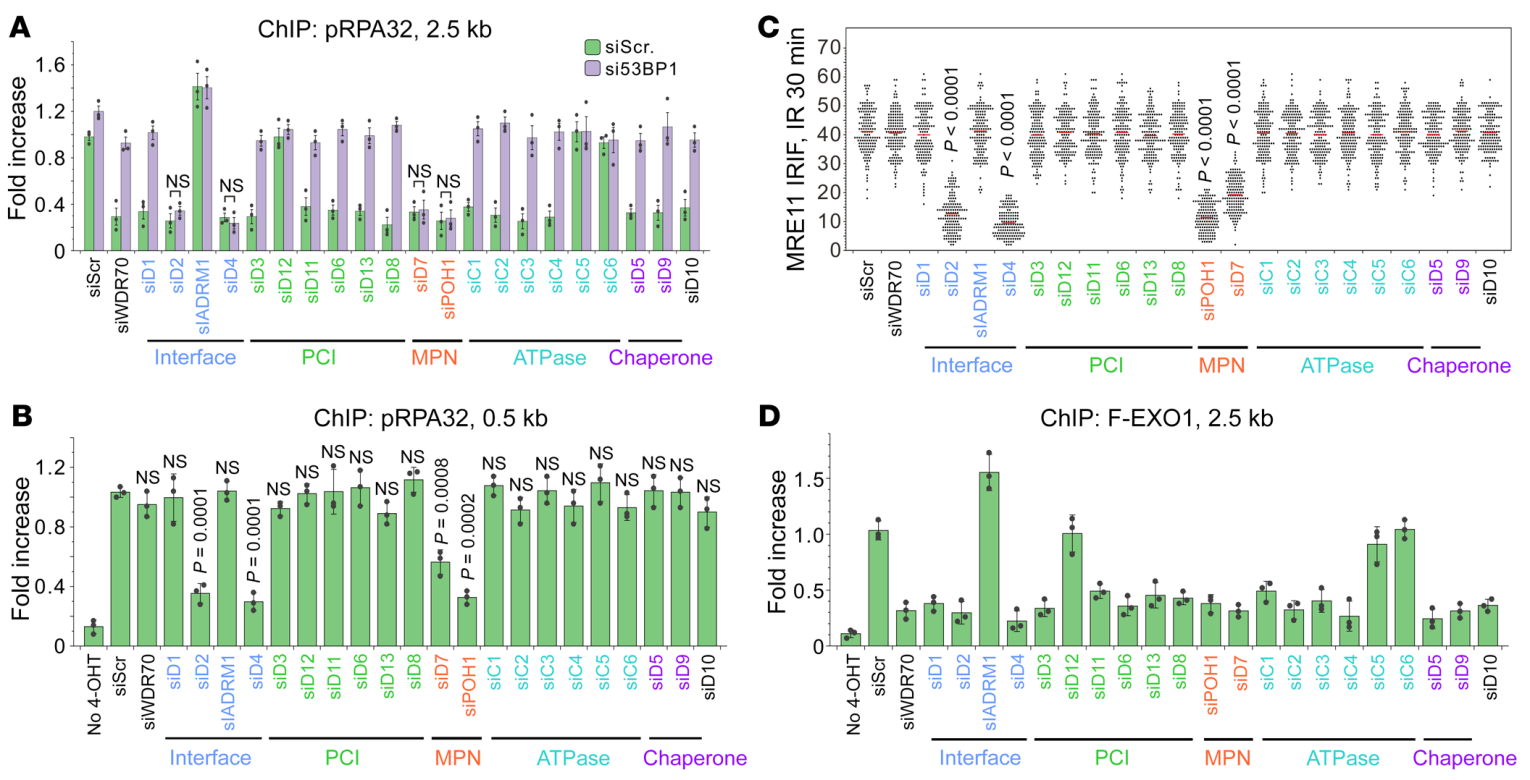
Separate CDW19S modules regulate MRE11 and EXO1 activation. (**A** and **B**) ChIP assay showing DSB loading of p-RPA32 at 2.5 kb (**A**) or 0.5 kb (**B**) distal to an *Asi*SI-induced DSB upon silencing of indicated CDW19S subunits. Concomitant *53BP1* knockdown was performed in **A**. (**C**) Enumeration of MRE11 foci upon silencing of individual CDW19S subunits in RPE1 cells. Immunofluorescence was carried out 30 minutes after IR. *n* = 3 biological repeats, 50 cells counted for each repeat. Error bars indicate SD. *P* values by *t* test are shown. (**D**) ChIP assay showing loading of EXO1 at 2.5 kb distal to an *Asi*SI-induced DSB.

Long-range resection is mainly promoted by exonuclease 1 (EXO1) and suppressible by 53BP1. To establish whether CDW19S has a functional domain mediating extensive resection, individual subunits were knocked down, and p-RPA32 recruitment was assayed 0.5 and 2.5 kb from an *Asi*SI site following 4-OHT induction. As seen with si*WDR70*, ablation of the majority of 19S components led to p-RPA32 reduction at 2.5 kb. For many components, this was effectively reverted by concomitant si*53BP1* treatment (Figure 3A). The effects of si*PSMD4^Rpn10^* and si*PSMD5^Hsm3^* on RPA32 loading were reproduced with a second siRNA, and each of these was complemented by respective siRNA-resistant plasmids (Supplemental Figure 3A). Interestingly, the distal resection defects observed upon *PSMD2^Rpn1^*, *PSMD4^Rpn10^*, and MPN (*POH1^Rpn11^* and *PSMD7^Rpn8^*) depletion were not restored by si*53BP1*. This correlates to inability of *53BP1* knockdown to rescue p-RPA32 reduction at 0.5 kb of these mutants (Figure 3B and Supplemental Figure 3B), documenting the role of these 4 components in initiating proximal resection that does not require CRL^WDR70^ or RP subunits with *WDR70*-like phenotypes.

To further explore these distinctions, we analyzed the formation of MRE11 IRIF. CRL4^WDR70^ depletion or *HBx* expression does not diminish the proximal nuclease (MRE11) from DNA ends (5). However, the ablation of *PSMD2^Rpn1^*, *PSMD4^Rpn10^*, *PSMD7^Rpn8^*, or *POH1^Rpn11^* prevented the formation of MRE11 IRIF 30 minutes and 2 hours after IR (Figure 3C and Supplemental Figure 3, C and D). This reflects their requirement for both proximal (0.5 kb) and distal (2.5 kb) processing (Figure 3, A and B). We therefore categorize an MRE11-regulatory module (MRM) within CDW19S that is necessary to initiate end processing.

We next analyzed chromatin association of the long-range exonuclease EXO1. Distal EXO1 association (2.5 kb) was reduced upon silencing of *WDR70* and CDW19S subunits that phenocopy *WDR70* (Figure 3D). The same impact was not observed upon silencing *ADRM1^Rpn13^* and non-functional components including *PSMD12^Rpn5^*, *PSMC5^Rpt6^*, or *PSMC6^Rpt4^*. Again, that si*ADRM1^Rpn13^* promotes distal EXO1 and p-RPA32 recruitment relative to control cells suggests that ADRM1^Rpn13^ limits, rather than promotes, long-range resection (Figure 2D and Figure 3, A and D). We conclude that CRL4^WDR70^, together with most RP components, constitutes an EXO1-specific module (ESM) that regulates extensive resection.

### PSMD1^Rpn2^, ADRM1^Rpn13^, PSMD5^Hsm3^, and PSMD9^Nas2^ are required for CRL4^WDR70^ recruitment.

**Figure 4:**
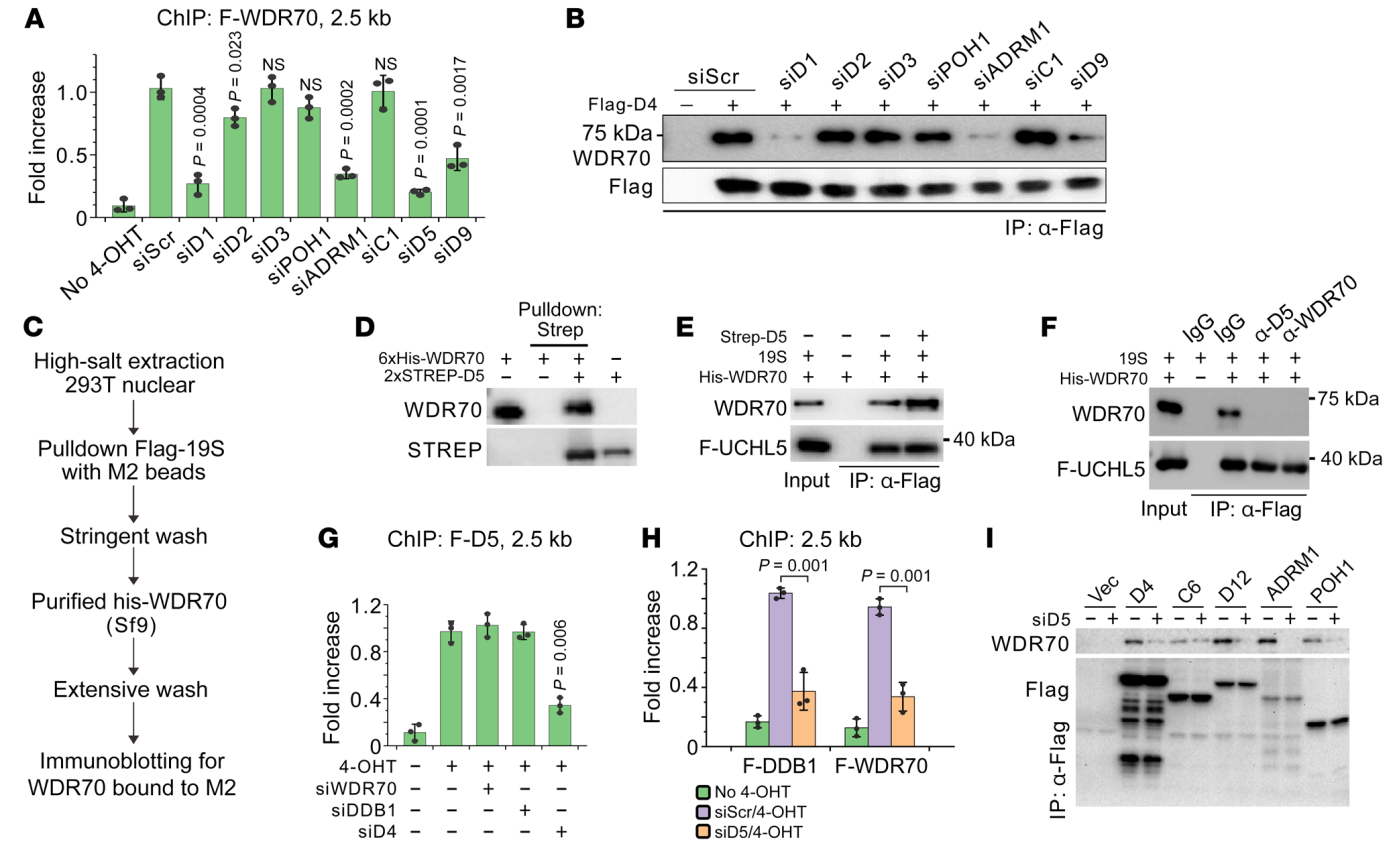
The docking platform of CRL4^WDR70^ on 19S RP. (**A**) ChIP assay for FLAG-tagged WDR70 at 2.5 kb distal to an *Asi*SI-induced DSB following siRNA treatment of 19S subunits. Data normalized to si*Scramble* with 4-OHT induction. (**B**) Coimmunoprecipitation of FLAG-PSMD4^Rpn10^ and WDR70 from chromatin fractions of CPT-treated HEK293T cells with or without ablation of indicated RP components. (**C**) Schematic showing the high-salt procedure for screening 19S components mediating direct engagement with WDR70. (**D**) Pull-down assay using purified WDR70 and PSMD5^Hsm3^. (**E** and **F**) In vitro pull-down assay for purified WDR70 (0.5 μg) and 19S proteasome (2 μg), the latter containing FLAG-UCHL5. Recombinant PSMD5^Hsm3^ (1 μg, **E**) or specific antibodies (0.5 μg, **F**) were added into the reaction. (**G** and **H**) ChIP assay for loading of FLAG-tagged PSMD5^Hsm3^ (**G**) or DDB1/WDR70 (**H**) 2.5 kb distal to an *Asi*SI-induced DSB following siRNA treatments. (**I**) Immunoprecipitation for endogenous WDR70 and FLAG-tagged 19S subunits with or without *PSMD5^Hsm3^* silencing.

To establish how CRL4^WDR70^ docks to the RP, WDR70 ChIP was exploited in the DIvA system combined with targeted siRNA. CRL4^WDR70^ loading at 2.5 kb following *Asi*SI nuclear import was significantly compromised by PSMD1^Rpn2^, ADRM1^Rpn13^, PSMD5^Hsm3^, and PSMD9^Nas2^ ablation (Figure 4A), implying that a platform dependent on these 4 ESM subunits tethers CRL4^WDR70^ to the RP. Ablation of other subunits of MRM (PSMD2^Rpn1^, POH1^Rpn11^) and ESM (PSMD3^Rpn3^, PSMC1^Rpt2^, POH1^Rpn11^) did not exhibit dramatic impact. Further, coimmunoprecipitation between WDR70 and FLAG-PSMD4^Rpn10^ in chromatin fractions was abolished in the absence of PSMD1^Rpn2^, ADRM1^Rpn13^, and PSMD9^Nas2^, but was maintained upon depletion of representative interface (*PSMD2^Rpn1^*), PCI (*PSMD3^Rpn3^*), MPN (*POH1^Rpn11^*), or ATPase (*PSMC1^Rpt2^*) subunits (Figure 4B).

To identify a direct docking site of CRL4^WDR70^, 19S particles were dissociated in nuclear HEK293T extracts using high-salt buffer (600 mM NaCl) and incubated with His-WDR70 (112–654 aa) purified from Sf9 cells (Figure 4C). The sole putative WDR70 binding partner identified was PSMD5^Hsm3^, a chaperone that contributes to RP assembly and only loosely associates with mature 19S (34). This interaction was confirmed by coprecipitation of recombinant His-WDR70 and Strep-PSMD5^Hsm3^ (Figure 4D and Supplemental Figure 4A). Recombinant WDR70 also displayed affinity with purified 19S RP (R&D Systems, E-367) containing FLAG-UCHL5 (Figure 4E and Supplemental Figure 4B). This WDR70-19S interaction is attributable to a low amount of PSMD5^Hsm3^ that copurified with 19S (Supplemental Figure 4C), since it was boosted by the addition of additional (0.5 μg) recombinant PSMD5^Hsm3^ and was weakened by pretreatment with specific antibodies against either PSMD5^Hsm3^ or WDR70 (Figure 4, E and F). As with other CDW19S subunits, PSMD5^Hsm3^ deposition at DSB sites required PSMD4^Rpn10^ but not WDR70 or DDB1 (Figure 4G), and si*PSMD5^Hsm3^* impeded the DSB association of both DDB1 and WDR70 (Figure 4H). si*PSMD5^Hsm3^* also attenuated coimmunoprecipitation of endogenous WDR70 with FLAG-PSMD4^Rpn10^, PSMD12^Rpn5^, ADRM1^Rpn13^, and POH1^Rpn11^ (Figure 4I). PSMC6^Rpt4^ was mildly affected. Thus, PSMD5^Hsm3^, together with ADRM1^Rpn13^, PSMD1^Rpn2^, and PSMD9^Nas2^, chaperones CRL4^WDR70^ to the RP, reminiscent of the chaperone contribution to the stepwise assembly of RP base subcomplexes (34).

Hereby, we posit that PSMD4^Rpn10^ launches the DSB assembly of CDW19S, triggering proximal and distal resection via separate modular functions of MRM and ESM by regulating nuclease activities of MRE11 and EXO1. Commitment to long-range resection requires the engagement of CRL4^WDR70^ to ESM via PSMD5^Hsm3^ (Supplemental Figure 4D).

### CRL4^WDR70^ regulates the ubiquitin-dependent degradation of break-associated ADRM1^Rpn13^.

CRL4^WDR70^ is a ubiquitin ligase that promotes resection, suggesting that it targets an anti-resection factor for degradation and implicating that such a factor would be more abundant on broken chromatin in the absence of CDW19S. ADRM1^Rpn13^ is excessively associated with chromatin in the absence of CDW19S (see Figure 2E), and its ablation results in increased resection (see Figure 2D) and RPA/EXO1 loading (see Figure 3, A and D). We therefore tested ADRM1^Rpn13^ recruitment to an *Asi*SI-induced DSB by ChIP, with or without siRNA of *WDR70*. ADRM1^Rpn13^ was recruited more abundantly upon si*WDR70*, particularly at the distal (2.5 kb) region (Supplemental Figure 5A). Importantly, co-depletion of *ADRM1^Rpn13^* and *WDR70* restored EXO1 recruitment at 2.5 kb when compared with *WDR70* depletion alone (Supplemental Figure 5B).

**Figure 5:**
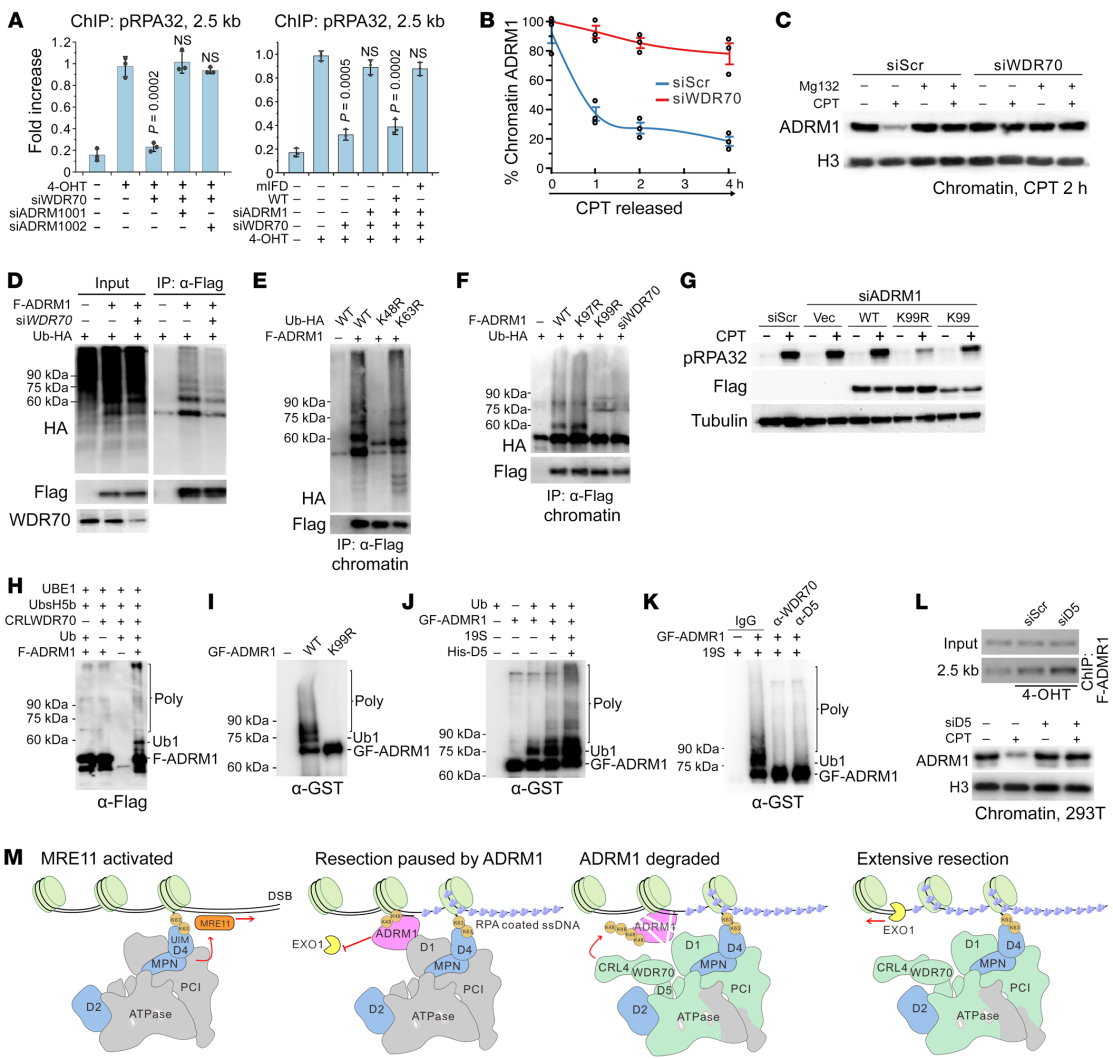
CRL4^WDR70^ targets ADRM1^Rpn13^ for UPS degradation. (**A**) Left: ChIP assay showing DSB loading of p-RPA32 at 2.5 kb distal to DSB upon silencing of *ADRM1^Rpn13^* (2 different siRNAs) or *WDR70*. Right: Equivalent assay expressing si001-resistant wild-type (WT) or m*IFD* mutant *ADRM1^Rpn13^*. (**B**) Protein abundance of ADRM1^Rpn13^ in chromatin fraction of 293T cells as measured by immunoblotting (see also Supplemental Figure 5E). *WDR70* was ablated, followed by CPT insult (2 μM) for 1 hour and release into drug-free medium. (**C**) Immunoblotting for ADRM1^Rpn13^ in cells cotreated with CPT and MG132. (**D**–**F**) Ubiquitin pull-down assay to identify polyubiquitinated species of FLAG-ADRM1^Rpn13^ upon treatment with si*WDR70* (**D**), expression of ubiquitin variants (**E**), or ADRM1^Rpn13^ K>R mutants (**F**). Cells were challenged with 2 μM CPT for 2 hours. (**G**) Immunoblotting for CPT-induced p-RPA32 upon expression of WT, K99R, or K99-only mutant of FLAG-ADRM1^Rpn13^ that is si*ADRM1^Rpn13^* resistant. (**H**) Reconstitution of FLAG-ADRM1^Rpn13^ ubiquitination catalyzed by purified proteins. (**I**) Equivalent reconstitution using WT or *K99R* versions. GF-ADRM1, GST/FLAG-tagged ADRM1. (**J** and **K**) ADRM1^Rpn13^ ubiquitination reconstituted with addition of purified 19S and/or His-PSMD5^HSM3^ (**J**), or in the presence of anti-WDR70 or anti-PSMD5^HSM3^ (**K**). (**L**) ChIP assay for DSB-associated ADRM1^Rpn13^ (2.5 kb from an *Asi*SI-induced DSB; top panel) and immunoblotting for chromatin-bound ADRM1^Rpn13^ following CPT treatment with or without *PSMD5^HSM3^* silencing (bottom panel). (**M**) Schematic for chromatin regulation of DSB repair by coordinative action of CDW19S modules.

Like PSMD4^Rpn10^, ADRM1^Rpn13^ is an RP ubiquitin receptor (35), and its DSB recruitment occurs independently of CDW19S (see Figure 2E). However, ADRM1^Rpn13^ supports the loading of WDR70 as part of the recruiting platform (see Figure 4A), implying their proximity. ADRM1^Rpn13^ encodes a C-terminal DEUBAD domain (265–407 aa) and a conserved N-terminal pleckstrin-like receptor for ubiquitin (Pru) (1–150 aa; ref. 35) that preferably binds to K48 polyubiquitin chains via a triple-residue motif. Substitution of *I75R*, *F76R*, and *D79N* (ADRM1^mIFD^) abolished the ubiquitin affinity (Supplemental Figure 5C), and impaired ADRM1^Rpn13^ enrichment at *Asi*SI-dependent DSBs (Supplemental Figure 5D). Two different *ADRM1^Rpn13^* siRNAs restored resection in the absence of *WDR70*, as measured by p-RPA32 ChIP (Figure 5A, left). Coexpression of siRNA-resistant wild-type *ADRM1^Rpn13^* abolished the rescue, whereas the m*IFD* version did not (Figure 5A, right), suggesting that ubiquitin association via the Pru domain is required for the anti-resection function. Therefore, CRL4^WDR70^ counteracts ADRM1^Rpn13^, and loss of ADRM1^Rpn13^ function obviates the need for CRL4^WDR70^ to promote extensive resection.

To further test whether ADRM1^Rpn13^ was being targeted for degradation via CRL4^WDR70^, stability of chromatin-associated ADRM1^Rpn13^ was evaluated. ADRM1^Rpn13^ was depleted from the chromatin fraction in CPT-challenged 293T cells, but remained stable when cotreated with si*WDR70* (Figure 5B and Supplemental Figure 5E) or proteasomal inhibitor (MG132; Figure 5C), revealing a WDR70-dependent and ubiquitin-proteasome system–dependent (UPS-dependent) turnover. Furthermore, polyubiquitination of chromatin-associated ADRM1^Rpn13^ was decreased upon si*WDR70* (Figure 5D) and impaired by the expression of a *K48R*, but not a *K63R*, ubiquitin mutant (Figure 5E).

Seven lysine residues conserved between human and yeast ADRM1^Rpn13^ (*Schizosaccharomyces* *pombe* has 2 homologs, Rpn13a and Rpn13b) were identified as putative ubiquitination targets (Supplemental Figure 5F). Surveying ubiquitination profiles in cells coexpressing individual FLAG-tagged K>R mutations and HA-tagged ubiquitin identified that mutation of *K99* (ADRM1^K99R^), but not other lysine residues (i.e., K97), abrogated ubiquitin-conjugated ADRM1^Rpn13^ species in the chromatin fraction (Figure 5F). Strikingly, an ADRM1^K99-only^ mutant was sufficient for ADRM1^Rpn13^ polyubiquitination, and this remained *WDR70* dependent (Supplemental Figure 5G). Consistent with K99 determining ADRM1^Rpn13^ ubiquitination, CPT-induced p-RPA32 level was reduced when si*ADRM1^Rpn13^* was complemented with ADRM1^K99R^ relative to that seen with either wild-type or ADRM1^K99-only^ (Figure 5G). Collectively, these results support the hypothesis that CRL4^WDR70^ promotes the ubiquitination and degradation of chromatin-bound ADRM1^Rpn13^.

### 19S complex boosts ADRM1^Rpn13^ ubiquitination via CRL4^WDR70^-PSMD5^HSM3^ engagement.

To corroborate the direct ADRM1^Rpn13^ ubiquitination by CRL4^WDR70^, the CRL4^WDR70^ E3 ligase was reconstituted using purified subunits (His-tagged CULLIN4A, DDB1, ROC1, and truncated WDR70^112–654^) coexpressed in Sf9 insect cells (Supplemental Figure 5H). The artificial CRL4^WDR70^ complex was supplemented with a ubiquitin reconstitution system (activating enzyme [E1], conjugation factor [E2, UbcH5b], and biotinylated ubiquitin) and tested for in vitro ubiquitinylation of bacterially expressed FLAG-ADRM1^Rpn13^ (Supplemental Figure 5I). Ubiquitin conjugates were observed with slow-migrating mono- and polyubiquitination species (Figure 5H), absent in control reaction conducted with ADRM1^K99R^ (Figure 5I). Consistent with the in vivo modification, ADRM1^Rpn13^ conjugates were K48-linked and were catalyzed by UbcH5b, but not UbcH5a or UbcH6 (Supplemental Figure 5, J and K).

In vivo, the loading platform containing ADRM1^Rpn13^ and PSMD5^Hsm3^ potentially juxtaposes the CRL4^WDR70^ E3 ligase to substrates (i.e., ADRM1^Rpn13^). We thus examined how purified 19S affected CRL4^WDR70^-dependent ADRM1^Rpn13^ ubiquitination. Ubiquitination activity was substantially stimulated by supplementing of RP particles to the CRL4^WDR70^-ADRM1^Rpn13^ (GST/FLAG-tagged) reconstitution system. This was further boosted by concomitant addition of recombinant His-PSMD5^Hsm3^ (Figure 5J) and inhibited by inclusion of anti-PSMD5 or anti-WDR70 antibodies (Figure 5K). Consistent with this, in vivo si*PSMD5^Hsm3^* resulted in accumulation of ADRM1^Rpn13^ at an *Asi*SI-induced DSB and stabilization after CPT treatment (Figure 5L). Taking together the results from Figures 2–5, we propose that ADRM1^Rpn13^ pauses end resection at DSB proximity, a function that relies on PSMD4^Rpn10^ and MRM. Additionally, we propose that to activate EXO1 and extensive resection, CRL4^WDR70^ acts as an RP-associated E3 ligase to catalyze UPS-mediated removal of ADRM1^Rpn13^, a function sustained by the *PSMD5^Hsm3^*-containing subdomain within ESM (Figure 5M).

### HBx attenuates the ESM to generate a viral HRD.

**Figure 6:**
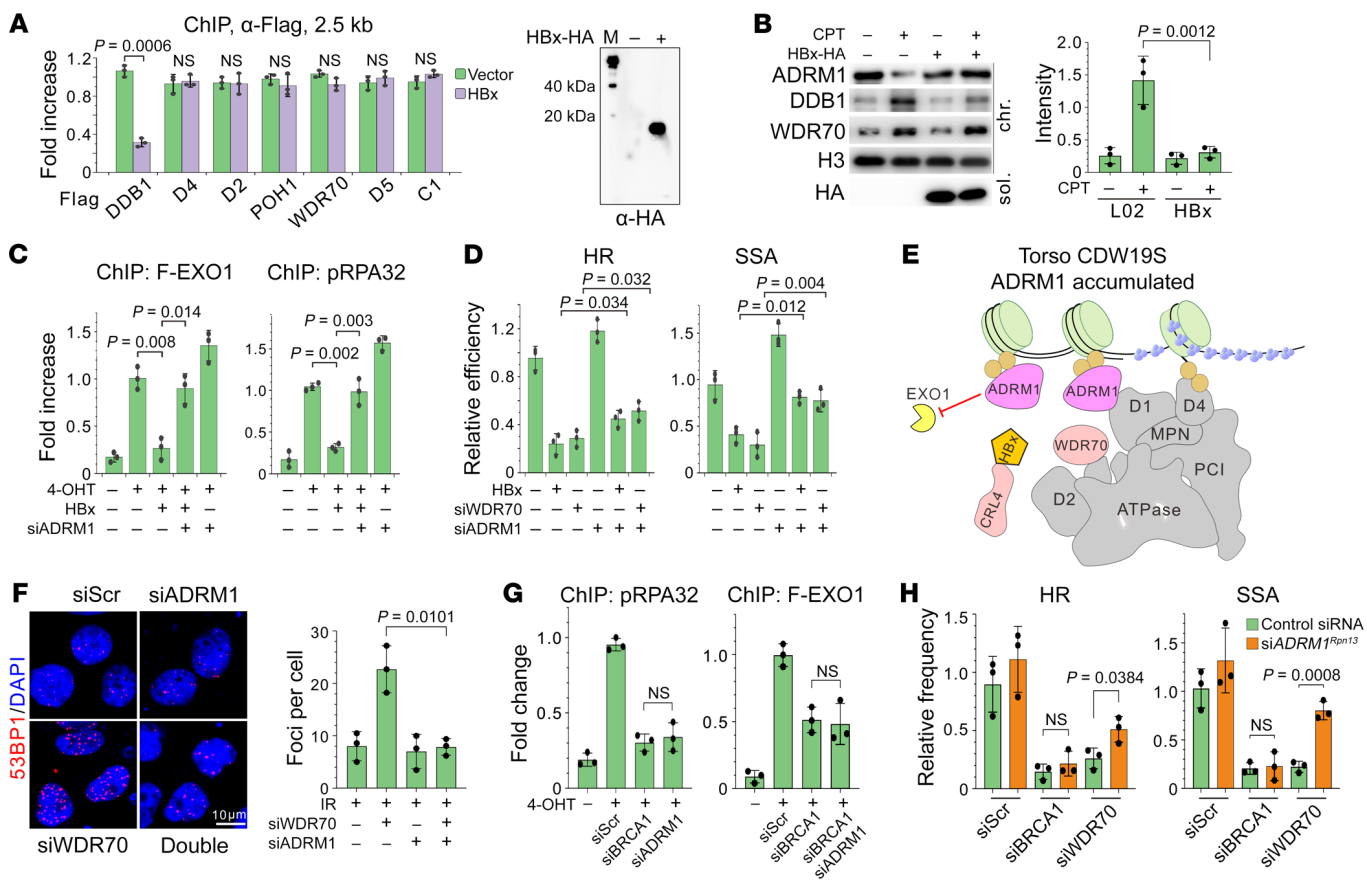
Torso CDW19S and ADRM1^Rpn13^ accumulation marks HBV-induced HRD subtype. (**A**) ChIP assay (left) for FLAG-tagged CDW19S subunits 2.5 kb from DSB in the presence or absence of HA-tagged *HBx* expression (right). Quantification was normalized to DDB1 value without *HBx* expression. (**B**) Chromatin and soluble nuclear fractionation of indicated proteins upon CPT insult with or without HBx-HA expression. Densitometry for DDB1 from 3 repeats is shown on the right. Results were obtained from identical biological samples immunoblotted from different concentrations of PAGE gels. (**C**) Enrichment of EXO1 or p-RPA32 loading 2.5 kb distal from DSB with *HBx* expression or si*ADRM1^Rpn13^*. (**D**) HR/SSA repair assay in the presence of *HBx* or si*WDR70* in L02 cells, with or without concomitant si*ADRM1^Rpn13^* treatment. (**E**) Schematic showing the HBx-induced “torso” CDW19S and consequent failure of ADRM1^Rpn13^ removal. (**F**) Representative images (left) and counting (right) of 53BP1 IRIF (8 hours after IR) in *WDR70*-ablated cells. Simultaneous *ADRM1^Rpn13^* silencing was performed as indicated. Scale bar: 10 μm. (**G**) ChIP showing the inability of si*ADRM1^Rpn13^* to restore the DSB loading of p-RPA32 and EXO1 in *BRCA1*-depleted cells. (**H**) Parallel comparison of HR/SSA improvement by control siRNA (green) or si*ADRM1^Rpn13^* (orange) in *BRCA1*- and *WDR70*-depleted cells. *P* values for multiple-group comparison in **B**–**D**, **F**, and **G** were calculated by 2-way ANOVA test.

HBx disintegrates the CRL4^WDR70^ complex by displacing WDR70 from CUL4A-DDB1 via a biomimetic H-box motif (5, 36). In HBV-integrated T43 hepatocytes PSMD2^Rpn1^ maintained its association with WDR70 while partially dissociating from DDB1, in contrast to its interaction with both proteins in HBV^–^ L02 cells (Supplemental Figure 6A). Given that DDB1 association with damaged chromatin is WDR70 dependent (4), we reasoned that HBx detaches the CRL4-DDB1 scaffold from ESM, leaving partially assembled CDW19S (WDR70-19S) at breaks. Indeed, expressing *HBx* in DIvA cells pronouncedly reduced DDB1 loading at *Asi*SI-induced DSBs without affecting other CDWS19 components (Figure 6A).

As a result, HBx impedes the EXO1-specific function of CDW19S. *HBx* expression in L02 cells accumulated chromatin-bound ADRM1^Rpn13^ but decreased DDB1 association upon CPT treatment (Figure 6B), an observation reproducible in another HBV^+^ HepG2.2.15 cell line relative to its parental line (HepG2; Supplemental Figure 6B). *HBx* reduced attachment of p-RPA32 and EXO1 distal (2.5 kb) to an *Asi*SI-induced DSB (Figure 6C), and this was not exacerbated when *PSMD4^Rpn10^* (ubiquitin-dependent DSB anchorage) or *PSMD5^Hsm3^* (ESM subunit) was concomitantly depleted (Supplemental Figure 6C). As with *WDR70* deficiency, si*ADRM1^Rpn13^* reversed the *HBx*-induced impediment of p-RPA32/EXO1 loading. As we reported previously, HBx does not affect MRE11 kinetics (5). Thus, HBx leaves “torso” CDW19S complex lacking the CUL4A-DDB1 scaffold at DSBs, and affects extensive resection by disrupting CRL4^WDR70^-containing ESM.

The impact of ADRM1^Rpn13^ accumulation and torso CDW19S was further evaluated by analysis of the repair outcomes in I-*Sce*I–induced DSB system. Imbalanced activities of NHEJ/HR in the presence of HBV or *HBx* were epistatic with siRNA of either *PSMD4^Rpn10^* or *PSMD5^Hsm3^* (Supplemental Figure 6, D and E). As expected, the HBx-dependent defect in pathway choice (HR and particularly SSA) was, in part, reversed by si*ADRM1^Rpn13^* (Figure 6D). Again, *ADRM1^Rpn13^* inhibition corrected repair bias in HepG2.2.15 cells (Supplemental Figure 6F). Collectively, torso CDW19S resulting from HBx induces HR defects by maintaining DSB-bound ADRM1^Rpn13^ and impeding EXO1-dependent resection (Figure 6E).

### ADRM1^Rpn13^ accumulation distinguishes viral HRD from BRCAness HRD.

We continued to establish the functional link between ADRM1^Rpn13^ and 53BP1 regarding their roles in resection barrier. Foci analysis revealed that ADRM1^Rpn13^ depletion reduced 53BP1 IRIF in *WDR70*-defective cells (Figure 6F), whereas depletion of *53BP1* exerted no effect on DSB-associated ADRM1^Rpn13^ in si*WDR70* or *HBx*-expressing cells (Supplemental Figure 6G). Apparently, ADRM1^Rpn13^ acts upstream to erase the 53BP1-mediated resection barrier. Given the rescuing effects of si*53BP1* on HRD in the context of torso CDW19S, these results indicate that 53BP1 absence can bypass the demand of ADRM1^Rpn13^ degradation for the commitment of long-range resection. Unlike the ability of *53BP1* depletion to rescue BRCAness HRD (see Figure 1E), si*ADRM1^Rpn13^* failed to restore the distal loading of p-RPA32 and EXO1, and to reverse the HR/SSA defects in *BRCA1-*depleted cells (Figure 6, G and H). Thus, while viral HRD and *BRCA1*-defective HRD share the common defect of 53BP1 accumulation, only the viral subtype is driven by excessive ADRM1^Rpn13^ deposition at DSBs.

### Targeting HBV-induced HRD suppresses the disease progression of HBV-associated hepatocellular carcinoma.

**Figure 7:**
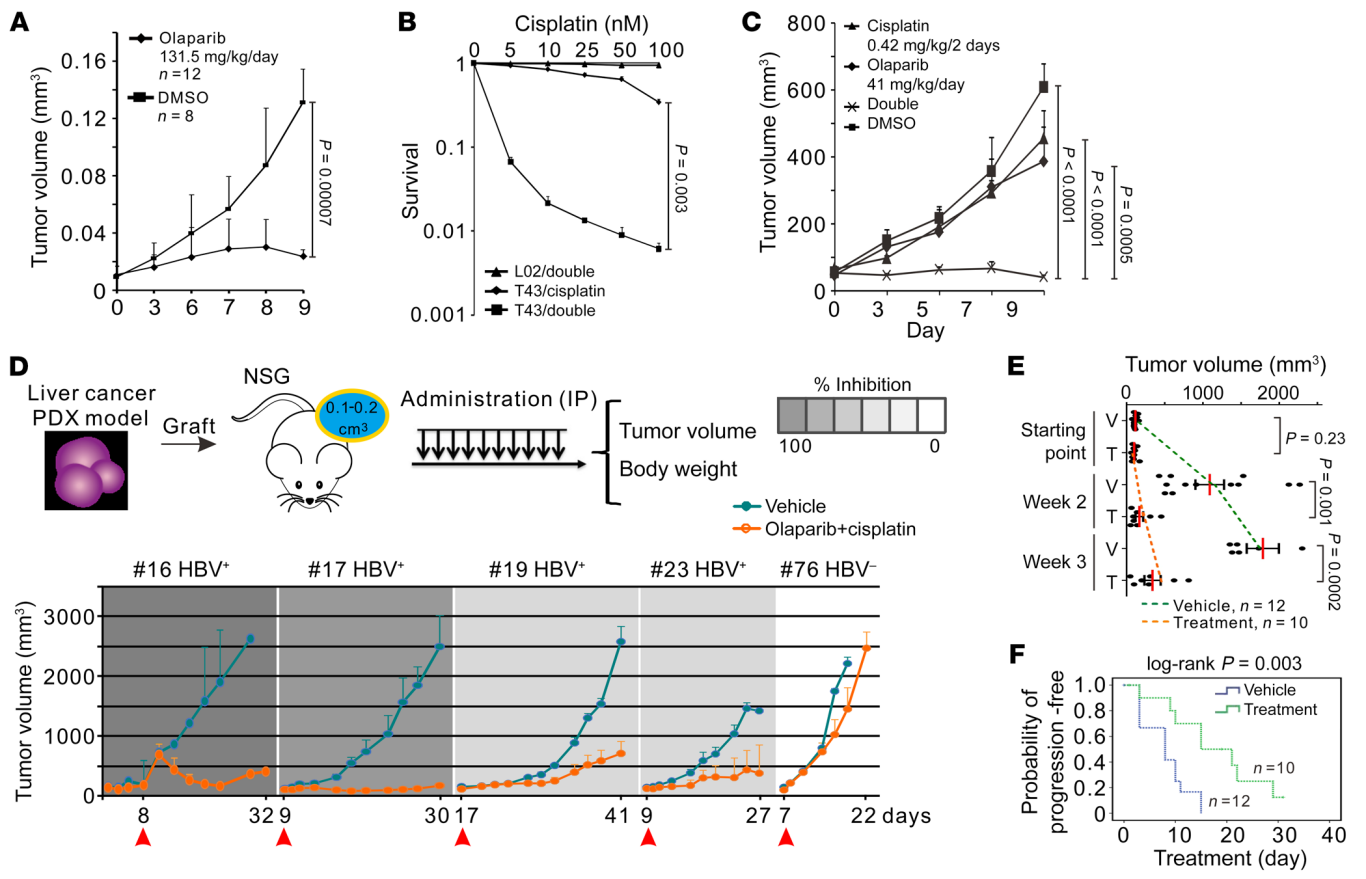
HBV-induced HRD subtype sensitizes HBVHCC to PARP inhibition. (**A**) Responses of T43 xenografts to monotreatment with olaparib or vehicle. Error bars indicate SD; *t* test. (**B** and **C**) Responses of T43 cells (**B**, 3 biological repeats) and xenografts (**C**, 6 littermates included) to conjunctive administration of olaparib and cisplatin. Tumor volumes are presented as means ± SD. DMSO: equivalent amount of solvent solution. Error bars indicate SD; *P* values were calculated by *t* test (**B**) and by 2-way ANOVA test (**C**). (**D**) Schematic of PARPi administration to HCC engraftment in NOD-SCID mice (top) and tumor responses (bottom). Tumor volumes of 4 HBVHCCs (patients 16, 17, 19, and 23) and 1 HBV-free HCC (patient 76, progressive disease) are shown at indicated days after inoculation. Olaparib: 33.3 mg/kg/d; cisplatin: 0.5 mg/kg/2 days (O/C). Horizontal axis, days after tumor transplantation; arrows, starting date of medication. Numbers of animals were as follows: patient 16 (vehicle, *n* = 4; treatment, *n* = 4), patient 17 (vehicle, *n* = 2; treatment, *n* = 2), patient 19 (vehicle, *n* = 3; treatment, *n* = 2), patient 23 (vehicle, *n* = 3; treatment, *n* = 2), patient 76 (vehicle, *n* = 3; treatment, *n* = 3). (**E**) Tumor response for HBVHCC patient-derived xenograft (PDX) sublines treated with vehicle or O/C at week 2–3. Graphs show mean ± SEM, analyzed with 2-sided unpaired Student’s *t* test. (**F**) Kaplan-Meier plot indicating progression-free survival of HBVHCC sublines. The *y* axis is the percentage of animals whose tumor volumes were smaller than 300 mm^3^. *P* value was calculated by log-rank test.

The overall survival of HBV-associated hepatocellular carcinoma (HBVHCC) patients is low, and few contemporary chemotherapeutic treatments are widely applicable. We exploited the viral HRD–induced SL and tested the potential of PARPi treatment in tumor-burdened immunodeficient mice. In athymic nude mice implanted with T43 xenografts, tumor growth was strongly restricted by 131.5 mg/kg/d olaparib monotreatment (Figure 7A and Supplemental Figure 7A). Notably, low doses of olaparib and cisplatin imposed synergistic effects on both cellular viability and T43 xenografts (Figure 7, B and C). To establish the benefits of PARPi treatment for HBVHCC, patient-derived xenografts were implanted in immunocompromised NOD*-Prkdc^scid^*-*IL2rg^(em1-IDOM)^* mice that subsequently were subjected to treatment with clinically relevant and mouse-equivalent dosage of olaparib (100 mg/kg/d) (37), in conjunction with a low dose of cisplatin (0.5 mg/kg/2 days). Cisplatin-insensitive hepatocellular carcinoma (HCC) xenografts from 4 HBV^+^ patients (patients 16, 17, 19, and 23) and 1 HBV-free patient (patient 76) were subjected to 1 course of olaparib/cisplatin (O/C) conjunctive treatment. Body weights in both O/C and vehicle groups were monitored throughout the experiment (Supplemental Figure 7B). Compared with the unrestricted tumor growth in the vehicle-only group, all HBVHCC cases treated with O/C displayed tumor growth inhibition (from 95.17% to 51.09%) at terminal therapy (Figure 7D), reflecting significantly delayed tumor progression (Figure 7E and Supplemental Figure 7C). Furthermore, O/C treatment produced a significantly longer median period of progression-free survival compared with that of vehicle groups (*P* = 0.003; Figure 7F). In contrast, xenografts derived from HBV-free HCC (patient 76) exhibited no therapy response when subjected to the same course of O/C treatment (Figure 7D).

## Discussion

In addition to regulating proteolysis, the 19S regulatory particle has a variety of non-proteolytic functions. Among these are a range of activities in the context of chromatin, including transcription initiation and elongation (18, 38, 39), roles in histone modification (40), and DNA repair (19–25). In this study we identify an RP decorated with CRL4^WDR70^ (CDW19S) that assembles in the vicinity of DSBs. We show that CDW19S can be segregated into distinct functional modules defined by ubiquitin anchorage (PSMD4^Rpn10^) and subdomains required for the resection activity of MRE11 (MRM) and EXO1 (ESM) nucleases. Their differential functions at proximal (0.5 kb) and distal (2.5 kb) indicate a central role of 19S RP in coordinating proximal and distal DNA end processing.

Mechanistically, we identified ADRM1^Rpn13^ as a direct catalytic substrate for CRL4^WDR70^ associated with the RP and show that preventing ADRM1^Rpn13^ degradation by mutating a single lysine residue (K99>R) attenuates distal resection, underpinning ADRM1^Rpn13^ as a key inhibitor of long-range resection upstream of 53BP1. Intriguingly, the equivalent K99 residue only exists in one of the fission yeast ADRM1 homologs, Rpn13b, and it was this protein that copurified with WDR70 (Figure 2A and Supplemental Figure 5F). This suggests a functional divergence between fission yeast Rpn13 paralogs in the context of UPS and 19S RP chromatin functions in low eukaryotes, while in human cells both functions are carried out by a single ADRM1^Rpn13^ version. We propose that CDW19S, upon association with RNF8-catalyzed K63 species, engages its associated enzymatic activities (i.e., CRL4^WDR70^) to remove the ADRM1^Rpn13^-dependent barrier and promotes long-range resection via EXO1 activation (Figure 5M).

Our uncovering of a pathological accumulation of ADRM1^Rpn13^ in the presence of HBV/HBx raises the intriguing question of how ADRM1^Rpn13^ functions in physiological contexts. In eukaryotes, resection is a vital process for HR and thus genome stability. However, end processing should also be tightly controlled to avoid overproduction of ssDNA structures (41) highly susceptible to nuclease attack (i.e., APOBEC/AID), as well as resulting mutagenesis (42, 43). From this angle, the pro- and counter-HR actions of CRL4^WDR70^-ADRM1^Rpn13^ constitute a quality control for appropriate DNA processing. Alternatively, as described in a recent study, 53BP1 may foster fidelity of homology-dependent repair by suppressing the switch from error-free gene conversion by RAD51 to mutagenic SSA by RAD52 (44). How ADRM1^Rpn13^ operates in these scenarios alongside the BRCA1-53BP1 control of pathway choice remains an interesting question.

The HBV oncoprotein HBx plays versatile roles during HBVHCC development, including redirecting the Cullin4-DDB1 scaffold to degrade SMC5/6 complex that counteracts viral replication (3). By hijacking the DDB1-containing ubiquitin ligases, HBx causes a deficit in the cellular ligases that rely on the Cullin4-DDB1 scaffold, including CRL4^WDR70^ and its associated function of HR (5). Here we clarify the underlying mechanism by which HBV/HBx pathogenesis causes HRD, showing that HBx prevents the integration of Cullin4-DDB1 into chromatin-associated CDW19S. This torso CDW19S is unable to promote ADRM1^Rpn13^ degradation, thus compromising homology-dependent repair. Notably, although this viral HRD subtype affects BRCA1-53BP1 function, it is different from canonical BRCAness HRD that does not involve the malfunction of ADRM1^Rpn13^.

BRCAness HRD renders cells sensitive to PARP inhibition as has been applied in breast cancer treatment (45, 46). In vitro studies suggest that HCC cell lines are also susceptible to PARP inhibition (47), corresponding to elevated levels of PARP1/2 enzymes and PARylation in HCC tissues (48, 49). While these studies did not indicate a clear HRD phenotype that deminishes the viability of HBVHCC cells through PARPi, we showed that HBV/HBx-induced viral HRD is SL to PARP inhibition, which relied on the impairment of CDW19S. We further demonstrate, using xenograft models of T43 cells or patient-derived HBVHCC materials, that PARPi treatment effectively constrains tumor growth and improves survival. This underlines the potential for SL treatment of HBVHCC using HRD toxins.

For breast cancer, HRD rates are estimated by either BRCAness mutations or single-nucleotide variation (signature 3) via whole-genome sequencing (WGS) (50). It would be worthwhile to determine the incidence and genomic features of viral HRD by WGS, which would not only provide the biomarkers for PARPi treatment of HBVHCC, but also help discriminate the fingerprints between BRCAness and HBV HRD subtypes.

Taken together, our results identify the mechanism by which HBV causes a special HRD subtype, and establish that HBVHCC is an HRD cancer type susceptible to PARP inhibition. Our findings provide a mechanistic justification for targeting HBVHCC by SL.

## Methods

### Cell culture and reagents.

Human cell lines (HEK293T, *WDR70^KO^*, RPE1, DIvA, HepG2 and HepG2.2.15, L02, and T43) were maintained in culture media supplemented with 10% FBS. L02 and T43 cells were propagated from single clones. The STR profile and hepatic identity of L02 are shown in Supplemental Figure 8, and T43 cells with stably integrated HBV genomes were regularly selected in G418 (200 μg/mL) and examined for titration of HBs and HBe antigens by ELISA (5). RPE1 and DIvA were obtained from the cell bank of the Genome Damage Stability Centre, University of Sussex. L02, HEK293T, HepG2, and HepG2.2.15 were purchased from National Infrastructure of Cell Line Resource (Wuhan or Shanghai, China). All cell lines tested negative for mycoplasma contamination. Plasmid and siRNA transfections were carried out using Lipofectamine 3000 (Invitrogen, L3000015). DSBs in DIvA system were induced by 300 nM 4-OHT (MilliporeSigma, H7904) for 4 hours. For ATM kinase or proteasome inhibition, 10 μM KU55933 (Selleck, S1092) or 100 μM MG132 (Selleck, S2619) was used for pretreatment for 2 hours. Plasmids, Primers, antibodies, and siRNA used in this study are listed in Supplemental Tables 2–5.

### Plasmids.

For cloning of lentiviral vectors for expression of FLAG-tagged proteins, PCR fragments were inserted into the *Eco*RI site of pLVX-G-FLAG plasmid using the In-Fusion cloning kit (Clontech, 639650). Plasmids used in this study are listed in Supplemental Table 2.

### Evaluation of gene expression.

For detection of gene expression, 10^6^ cells were harvested and total RNA was extracted using NucleoZOL Reagent (Macherey-Nagel, 740404). cDNA was obtained from 1 μg RNA by Reverse Transcription System (Promega, A3500). Real-time quantitative PCR (qPCR) and semiquantitative PCR were performed on a Bio-Rad CFX96 Real Time System according to the instructions of qPCR Kits (Promega, A6001) with 3 technical replicates for each sample. For reverse transcriptase qPCR, the relative expression was calculated by fold change of target gene normalized to GAPDH/18S rRNA in each sample, and the experimental group was normalized to the average of the control. Semiquantitative PCR products were resolved by 2% agarose electrophoresis.

### Tandem affinity purification for Wdr70-associating proteins.

To purify proteins in complex with Wdr70 of *S.* *pombe*, 8 liters of *spWdr70* tandem affinity purification–tagged (TAP-tagged) or control strains were grown in YEP rich media (1% Yeast Extract [Solarbio, Y8020], 2% Peptone [Solarbio, P8450], 0.5% NaCl). The cell pellet was washed and resuspended in an equal volume of lysis buffer (25 mM Tris-HCl [pH 7.5], 15 mM EGTA, 15 mM MgCl_2_, 0.1% NP-40, 1 mM DTT, 0.1 mM NaF) supplemented with protease inhibitor (Complete Mini, Roche, 11836153001) plus DTT (1 μM) and subjected to mechanical beating with glass beads in a ribolyzer. The granular cell pellets and the powder of ground cells were frozen at –80°C and stored (up to 3 months) before use. Soluble extracts were clarified by centrifugation before binding to rabbit IgG-coated Dynabeads M280 (Invitrogen, 11203D), followed by extensive washing and cleavage by tobacco etch virus (TEV) protease. Purified Wdr70-associating components were resolved by 8%–20% gradient SDS-PAGE and stained with Brilliant Blue G-Colloidal Concentrate Kit (MilliporeSigma, B-2025).

Excised gel bands were washed with 25 mM NH_4_HCO_3_ containing 50% acetonitrile and dehydrated with 100% acetonitrile, followed by treatment with 10 mM DTT and incubation for 1 hour at 65°C. After cooling, gel samples were alkylated with 55 mM iodoacetamide for 45 minutes at room temperature. Samples were digested with trypsin (1:50, wt/wt) dissolved in 25 mM NH_4_HCO_3_ at 37°C overnight. Digested peptides were extracted with 5 mM octyl-β-d-glucopyranoside in 0.25% trifluoroacetic acid for 60 minutes at 37°C and directly applied onto the AnchorChip target (Bruker Daltonics), which was loaded with α-cyano-4-hydroxycinnamic acid (CHCA) thin-layer matrix. Mass spectra of extracted peptides for each sample were determined using Ultraflex MALDI-TOF/TOF mass spectrometry (Bruker Daltonics) in a positive ion reflector mode. The ion acceleration voltage was 25 kV. Both MALDI-TOF spectra and the MS/MS spectra were processed by FlexAnalysis 2.2 (Bruker) and Biotool 2.2 (Bruker) and automatically searched against the Swiss-Prot database using Mascot software (Matrix Science). Main parameters were set as follows: mass range from 800 to 4,000 Da; S/N ≥ 3.0; fixed modification, carbamidomethyl (Cys); variable modification, oxidation (Met); maximum number of missing cleavages, 1; MS tolerance, 50 ppm; and MS/MS tolerance, 0.7 Da.

### Protein purification and pull-down assay.

Purified 6xHis-WDR70 (112–654 aa) and CRL4-WDR70 tetraplex from Sf9 insect cells were purchased from HitGen. For PSMD5 purification, the coding sequence was amplified from a cDNA library and cloned into the pET28a-2xStrep plasmid. For bacterial expression, BL21 strain transformed with expression vector was induced with 1 mM IPTG overnight at 16°C. Five hundred milliliters of cells were pelleted and resuspended in 20 mL TBST buffer with protease inhibitor cocktail and sonicated. Centrifuged supernatant was incubated with MagStrep XT Beads (IBA Lifesciences, 2-4090-002) and eluted with BXT buffer (100 mM Tris [pH 8.0], 150 mM NaCl, 1 mM EDTA, 50 mM biotin) for 1 hour. For ADRM1 wild-type or K99R purification, pET28a-FLAG plasmids were used with otherwise similar protocol except that incubation was with GSH beads (BeaverBeads, 70601-5) and elution with buffer B (50 mM Tris-HCl, 10 mM glutathione).

For immunoprecipitation, 10^6^ cells were harvested and lysed in 200 μL of buffer A (10 mM HEPES [pH 7.9], 10 mM KCl, 1.5 mM MgCl_2_, 0.34 M sucrose, 10% glycerol, 1 mM DTT, 0.1% Triton X-100, and protease inhibitors). Nuclei were collected in the pellet by low-speed centrifugation (1,500*g*, 4 minutes, 4°C) and further incubated with buffer B (2 mM EDTA, 0.2 mM EGTA, 1 mM DTT, and protease inhibitor mixture) for 10 minutes on ice. After centrifugation (2,000*g*, 4 minutes), the pellet was resuspended in IP2 buffer (50 mM Tris [pH 8.0], 150 mM NaCl, 1% NP-40, and protease inhibitor cocktail) with 100 U Micrococcal Nuclease (New England Biolabs, M0247S)/DNase I (New England Biolabs, M0303S) and incubated on ice for 30 minutes to digest genome DNA. The chromatin fraction was clarified by high-speed centrifugation (21,000*g*, 10 minutes) and then incubation with prewashed FLAG M2 beads (MilliporeSigma, M8823) to pull down FLAG-tagged immunocomplex. Proteins were detected by immunoblotting.

### Immunostaining.

Indirect immunofluorescent staining was described elsewhere (5). Briefly, cryosectioned tissues or cells on coverslips were fixed with 4% PFA. Permeabilized sections were incubated with primary antibodies and labeled with secondary antibodies (anti-rabbit–Cy3 or anti-mouse–FITC), followed by mounting with anti-fade medium containing DAPI (Vector Laboratories, H120010) and visualization using a Leica fluorescence microscope (DM4 M) or Olympus fluorescence microscope (BX51). All quantitative immunostaining analysis was performed by counting of 100–200 cells from 3 independent experiments.

For measurement of 53BP1 exclusion from the core IRIF, images were processed using FV10-ASW 3.1 Viewer (Olympus) software, and the cavity of 53BP1 and sizes of p-RPA32 foci were gauged by measurement of pixel densities by ImagePro Plus 6.0 (Media Cybernetics) across the center lines of foci with best visualization. Fifty to two hundred foci were measured for each group.

### In vitro ubiquitination assay.

Recombinant E2 enzymes (R&D Systems, K-980B) and ubiquitin conjugation initiation kit (R&D Systems, K-995) were used for ubiquitination assay. In brief, 500 ng purified ADRM1 or mutant protein was incubated with UBE1, UbcH5b, ubiquitin, CRL4^WDR70^ tetramer (500 ng) at 37°C for 1 hour. The samples were mixed with SDS sample buffer and detected by immunoblotting following SDS-PAGE. Recombinant ubiquitin mutants K48R (R&D Systems, UM-K48R) and K63R (R&D Systems, UM-K63R) were used for identification of ubiquitin chain species. Recombinant 19S proteasome with FLAG-tagged UCHL5 was purchased from R&D Systems (E-367).

### Measurement for efficacies of DSB repair.

For plasmid-based DSB repair system (26), pCMV plasmids containing the I-*Sce*I restriction site were subject to thorough in vitro digestion. Cut plasmids (NHEJ and SSA: 5 μg; HR: 10 μg; gift from Jun Chen, Zhejiang University, Hangzhou, China) were transfected into 293T or WDR70-knockout cells and allowed to repair in vivo for 48 hours. Recombined or ligated plasmids by SSA/HR (primers 3/4) or NHEJ (primers 5/4) were recovered from cells by a HighPure PCR Template Preparation Kit (Roche, 11796828001), followed by quantitative real-time PCR with appropriate primers (Supplemental Table 3) specific for SSA/HR or NHEJ fragments using a Bio-Rad CFX96 Real Time System. The amount of extracted plasmid DNA was normalized to the product of primers 1/2. The relative frequency of each repair pathway was defined as fold changes relative to qPCR values of repaired DNA extracted in parallel from wild-type or mock-infected 293T cells.

For the *Xba*I-dependent resection assay, genomic DNA from 2 × 10^5^ DIvA cells following 4-OHT induction was purified using High Pure PCR Template Preparation (Roche, 11796828001). For each sample, 300 ng of extracted DNA was digested with *Xba*I at 37°C for 4 hours, and the reaction was stopped by heating at 65°C for 10 minutes. For ssDNA quantification, 20 ng digested sample was amplified by qPCR using primers listed in Supplemental Table 3.

### Chromatin immunoprecipitation.

Chromatin immunoprecipitation (ChIP) assays were performed as previously described with minor modifications (51). Briefly, for each location reaction, approximately 4 × 10^6^ cells were harvested and then cross-linked for 10 minutes with formaldehyde at a final concentration of 1% and washed twice in PBS. Cells were then lysed in lysis buffer (50 mM HEPES [pH 7.4], 140 mM NaCl, 1% Triton X-100, 0.1% Na deoxycholate, 1 mM EDTA supplemented with protease inhibitor cocktail [Roche, 11836170001]) and sonicated to solubilize chromatin and shear the cross-linked DNA. Sonication was performed at 4°C with Bioruptor Pico (Diagenode) at default power for 40 30-second long pulses (30-second pause between pulses). To retrieve chromatin-associated proteins, the whole-cell extracts were incubated on a rotator overnight at 4°C with 10 μL magnetic Protein G Dynabeads (Invitrogen, 10004D) precoated with 2 μg of the indicated antibodies or FLAG M2 beads (MilliporeSigma, M8823). The beads were washed sequentially for 5 min with lysis buffer (see above), lysis high-salt buffer (50 mM HEPES [pH 7.4], 500 mM NaCl, 1% Triton X-100, 0.1% Na deoxycholate, 1 mM EDTA), wash buffer (10 mM Tris [pH 8.0], 250 mM LiCl, 0.5% NP-40, 0.5% Na deoxycholate, 1 mM EDTA), and TE buffer (pH 8.0), and twice for each buffer. Bound DNA-immune complexes were eluted off the beads in elution buffer (50 mM Tris [pH 8.0], 1% SDS, 10 mM EDTA) by heating at 65°C for 2 hours. DNA was purified using HighPure PCR Template Preparation Kit (Roche, 11796828001). Whole-cell extracts were treated in parallel for cross-link reversal and DNA extraction. Two hundred nanograms recovered DNA was used for each PCR reaction using Platinum *Taq* DNA Polymerase (Invitrogen, 10966). All reactions were performed in triplicate. Primer sets used to measure protein enrichment were as follows: CRISPR system (input, pair 22/23; 0.5 kb, pair 6/7; 3.5 kb, pair 8/9; 10 kb, pair 18/19); DIvA system (0.5 kb, pair 26/27; 1 kb, pair 28/29; 2.5 kb, pair 30/31; 5 kb, pair 32/33).

### Preparation of metaphase chromosomal spread.

Cells were plated in a 60-mm dish and arrested in mitosis by 2-hour treatment with colcemid (final concentration of 200 ng/mL). Prewarmed 75 mM KCl was then added to trypsinized cells and incubated for 15 minutes at 37°C. Then 4 drops of freshly prepared fixative (3:1 solution of methanol/acetic acid) were added. Cells were pelleted and resuspended in 5 mL fixative and incubated for 20 minutes at 4°C. After the fixing step was repeated 3 or 4 times, cell pellets were resuspended in 0.5 mL fixative solution. Two or three drops of fluid were precipitated onto a prechilled slide from a height of 18 inches. Slides were air-dried thoroughly and stained using the Giemsa protocol. The mitotic chromosomes were observed and evaluated using an Olympus fluorescence microscope (BX51) at ×1,000 magnification.

### Cell survival assay.

For colony formation assays, cells were plated at 500 cells each in a 10-cm dish and cultured at 37°C for 10–14 days, then fixed with methanol and stained with Giemsa solution. PARPi (olaparib/KU0059436, talazoparib/S7048, Selleck; niraparib/MB5556, veliparib/MB5524, rucaparib/MB1643, Meilunbio) or nutlin (ApexBio, A4228) was applied in the indicated concentrations alone or simultaneously used with diluted cisplatin (Supertrack Bio-pharmaceutical, 131102). Cell proliferation of *WDR70*-knockdown 293T cells was measured by cell counting every other day after transfection.

### PARP inhibition using nude mice and xenograft model.

All animal experiments were carried out in animal facilities of West China Second University Hospital or at an outsourcing service (Beijing IDMO Co. Ltd.). Athymic nude immunodeficient mice (BALB/c nu/nu, specific pathogen free) were purchased from the animal center of Sichuan University. For T43 xenograft mouse models, female nude mice starting at 4–5 weeks of age were used for the experiments. To assess the tumorigenicity of L02 or T43 cells in vivo, mice were subcutaneously inoculated in both sides of armpits or hind flanks (4 × 10^6^ cells per site). When T43 xenografts had reached an average volume of approximately 0.01 cm^3^, animals were randomized (using a random number table) into treatment groups, and 5 animals were selected for each group. Monotreatment with olaparib was administered intraperitoneally to each animal at a dosage of 131.5 mg per kg body weight per day. For double treatment, olaparib was administered at a dosage of 41 mg per kg body weight per day, and cisplatin at 0.42 mg per kg body weight every 2 days. A parallel group of mice was given PBS-diluted DMSO as control. Inoculated and drug-administered mice were observed each day. Sizes of tumors were measured by Vernier caliper and tumor volumes calculated by 3D measurement (length × width × height × π/6) until termination of the experiments. To exclude outliers of tumor volume, Grubbs’s test was applied (Gi>GO. 95, 2-tailed test). Experiments were performed blindly.

Patient-derived xenograft assays were established and performed in specific pathogen–free facilities of Beijing IDMO Co. Ltd. Primary HBVHCC tissues were enzymatically dissociated, and primary tumor cells were subcutaneously inoculated into the flank of NOD-*Prkdc^scid^-IL2rg^(em1-IDOM)^* (NPI) recipient mice. After sufficient tumor growth, tumors were passaged or cryopreserved for banking. For PARPi treatment of HBVHCC, 4 cisplatin-insensitive HBVHCCs and 1 HBV-free HCC were inoculated subcutaneously to NSG mice at 4–6 weeks of age. Randomized and age-matched males were used with an even split between control and drug administration. In total, 22 tumor-burdened mice survived to the terminal stage of the experiments, and data points were collected from these animals. Each tumor included a vehicle and an experimental group, to which 2–3 repeats were allocated, performed blindly. Vehicle (12.5% DMSO in PBS) or combined olaparib (33.3 mg/kg/d) plus cisplatin (3 mg/kg/2 days) treatment was administered by intraperitoneal injection. The health of the animal was monitored daily throughout therapy. Sizes of xenograft implantations were regularly measured by Vernier caliper and calculated for 3D volumes. Mean percentages of inhibition were calculated by % inhibition = (mean(control) – mean(treatment))/mean(control) × 100%. HBV^+^ and HBV^–^ HCC materials used in patient-derived xenograft experiments were selected according to the HBV infection status: HBV^+^ tumors were serologically HBsAb^–^HBsAg^+^HBcAb^+^HBeAb^+^HBeAg^–^, and HBV^–^ tumors were HBsAb^+^HBsAg^–^HBcAb^–^HBeAb^–^HBeAg^–^. All HCCs were from Chinese male patients.

### Data analysis.

The percentage of tumor growth inhibition was defined as [1 – (mean volume of treated tumors)/(mean volume of control tumors)] × 100%. Progression-free survival (PFS) was defined in this study as the condition where tumor volumes were under 300 mm^3^. Mice with tumor sizes exceeding 300 mm^3^ were assigned “progressive disease” and taken off PFS calculation. These mice could be enrolled again into PFS calculation if tumor regressed to less than 300 mm^3^ during later treatment. The PFS curves were generated by the Kaplan-Meier method, and log-rank tests were used to compare PFS in each experimental group.

### Statistics.

All histograms are presented as means ± SD. For quantitative analysis including ChIP assay, image analysis, and repair analysis, at least 3 independent experiments were carried out. Kaplan-Meier plots and log-rank tests were computed by SPSS 16.0 software to compare the survival outcomes between 3 groups. *P* values were calculated by 2-tailed Student’s *t* test between 2 groups, or by 2-way ANOVA test for multiple-group comparison, using GraphPad Prism 6. *P* values less than 0.05 were considered significant. Endpoint values of cell survival and antitumor assays were used for statistical analysis.

### Study approval.

Animal studies were approved by ethical committees of West China Second University Hospital, Chengdu, China (Medical Research 2018(015)) and Beijing IDMO Co. Ltd., Beijing, China (P20211229001), and were performed strictly in compliance with the ethical guidelines and regulations. Animals were housed in accordance with approved protocols, and efforts were made to minimize suffering. Deidentified tumor material was collected from patients in agreement with institutional ethical regulations. Informed consent was provided via protocol [Medical Research 2018(014)] approved by the ethical committees of West China Second University Hospital.

### Data availability.

Data included in this article are provided in the Supporting Data Values file and are available upon request from the corresponding authors. Participant data are available upon request from CL (congliu@scu.edu.cn) under standard rules of data protection and ethical permissions.

## Author contributions

CL purified CDW19S complex, and MZ characterized it. LR and HW analyzed 53BP1 and BRCA1 in the absence of CRL4^WDR70^. JC assisted with I-*Sce*I repair assay. XW performed immunoblotting and cell culture. WZ, ZL, and JJ identified components by mass spectrometry. X Mo advised on animal models. ZT investigated PARPi toxicity and anticancer studies. X Mao and JH contributed CRL4^WDR70^-mediated ADRM1 degradation. DK, AMC, and CL designed and supervised the research and interpreted data. CL and AMC acquired funding and edited and reviewed the manuscript. Co–first authors were assigned based on the academic contribution of individual authors: characterization of CDW19S architecture and mechanism by MZ, exploration of targeted therapy by ZT and the establishment of CRL4WDR70 in 53BP1 removal (LR and HW).

## Supplementary Material

Supplemental data

Supporting data values

## Version 1 10/10/2023

In-Press Preview

## Version 2 12/01/2023

Electronic publication
