# Supplementary material for: Hepatitis B virus infection disrupts homologous recombination in hepatocellular carcinoma by stabilizing resection inhibitor ADRM1
Source: J Clin Invest. 2023 Dec 1;133(23):e171533. doi: 10.1172/JCI171533 (PMC10688980; doi:10.1172/JCI171533)
Supplement: Supplemental data [file jci-133-171533-s201.pdf]

Supplementary Figure

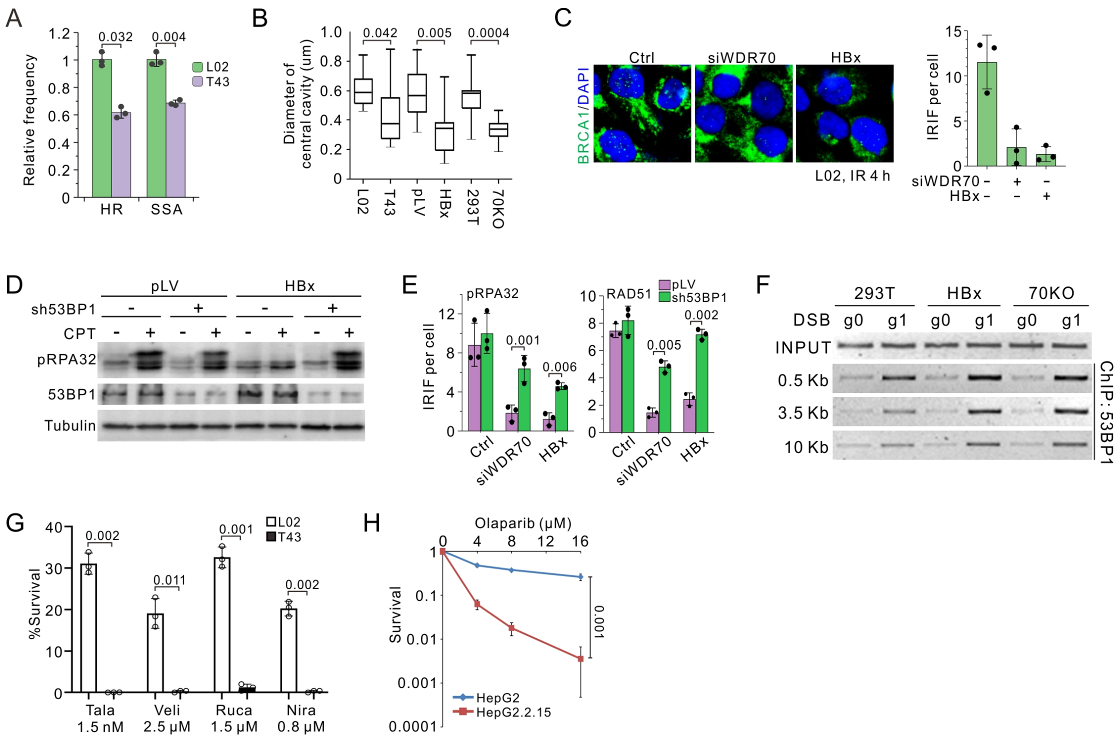

Supplemental Figure 1. HBx disturbs the CRL4^{WDR70}-dependent removal of resection barriers.

(A) Repair efficiencies by HR and NHEJ at an I-*Sce*I-induced DSB in HBV carrier cells (T43) were measured by real-time PCR relative to the parental cell line (L02).

(B) Range of 53BP1 cavity sizes (µm) in indicated cells measured as in Figure 1B. n = 3 biological repeats and 25 cavities were included for each sample. box: median and interquartile range; whiskers: minimum and maximum.

(C) Image and quantification for BRCA1 IRIF in L02 cells with indicated treatments.

(D) Immunoblot detection of pRPA32 after CPT treatment in *HBx* expressing L02 cells expressing, or not, sh*53BP1*.

(E) Quantification for pRPA32 and RAD51 IRIF in 293T cells treated with si*WDR70* or *HBx* expression. *53BP1* was knocked down by infecting sh*53BP1* lentivirus. pLV: vector control.

(F) ChIP assays depicting 53BP1 chromatin loading at indicated distance from the DSB upon expression of gRNA (g1) targeting the *PPP1R12C/p84* locus. g0: control gRNA.

(G) Percentages of T43 survival after challenging with indicated PARPi assayed by colony formation. n = 3 biological repeats; *p* values by *t*-test are shown; error bars: s.d.

(H) Viability of HepG2 (HBV-) and HepG2.2.15 (HBV+) upon Olaparib treatment.

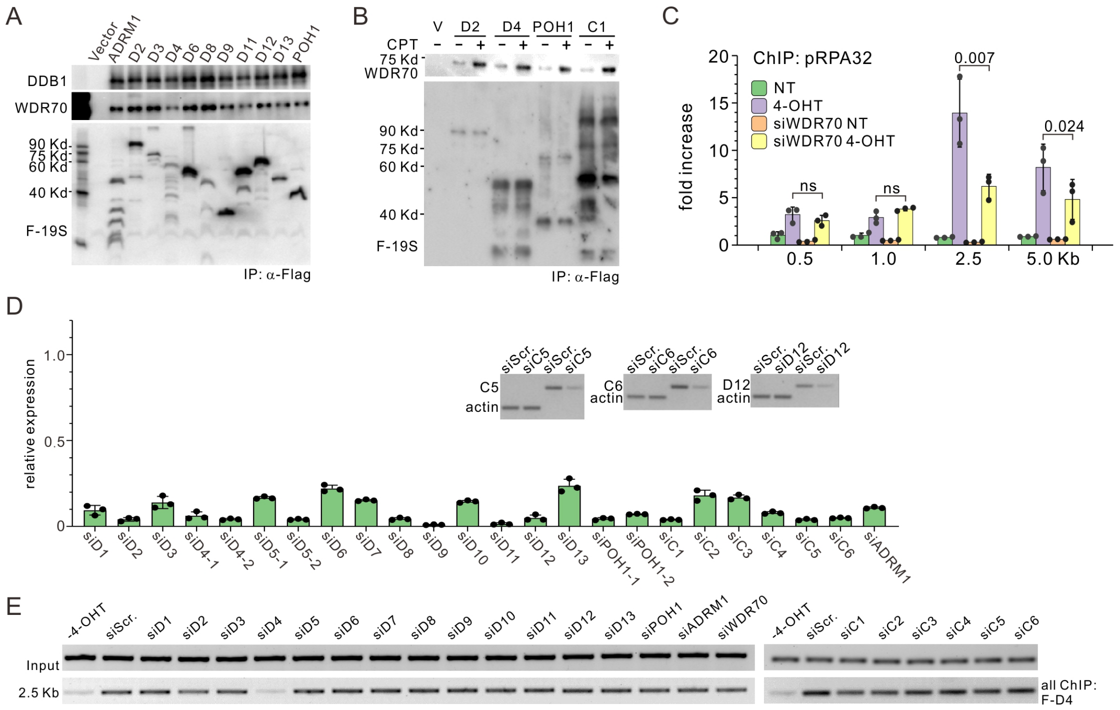

Supplemental Figure 2. The DSB assembly of CDW19S.

(A) co-IP of endogenous WDR70 and DDB1 in HEK293T nuclear extracts by Flag-tagged 19S subunits.

(B) co-IP of endogenous WDR70 by Flag-tagged PSMD2^{Rpn1}, PSMD4^{Rpn10}, POH1^{Rpn11} and PSMC1^{Rpt2} in chromatin extraction pre-treated with CPT (2 μM, 2 hours) or not.

(C) *Asi*SI-dependent DSB-association of pRPA32 assayed by ChIP 4 hours after 4-OHT addition. *WDR70* was depleted by siRNA. n=3 biological repeats, Error bars; s.d., *p* values are shown by two-way ANOVA test for multiple-groups comparison.

(D) Knockdown efficiencies in DIvA cells evaluated by RT-qPCR for individual 19S genes relative to si*Scramble* after normalization to 18S *rRNA* levels. Semi-quantitative PCR for *PSMC5*^{Rpt6}, *PSMC6*^{Rpt4} and *PSMD12*^{Rpn5} as well as actin controls are shown in inset. n=3 biological repeats. Error bars; s.d.

(E) ChIP assay for Flag-tagged PSMD4^{Rpn10} at 2.5 Kb from an *Asi*SI-induced DSB with indicated treatments.

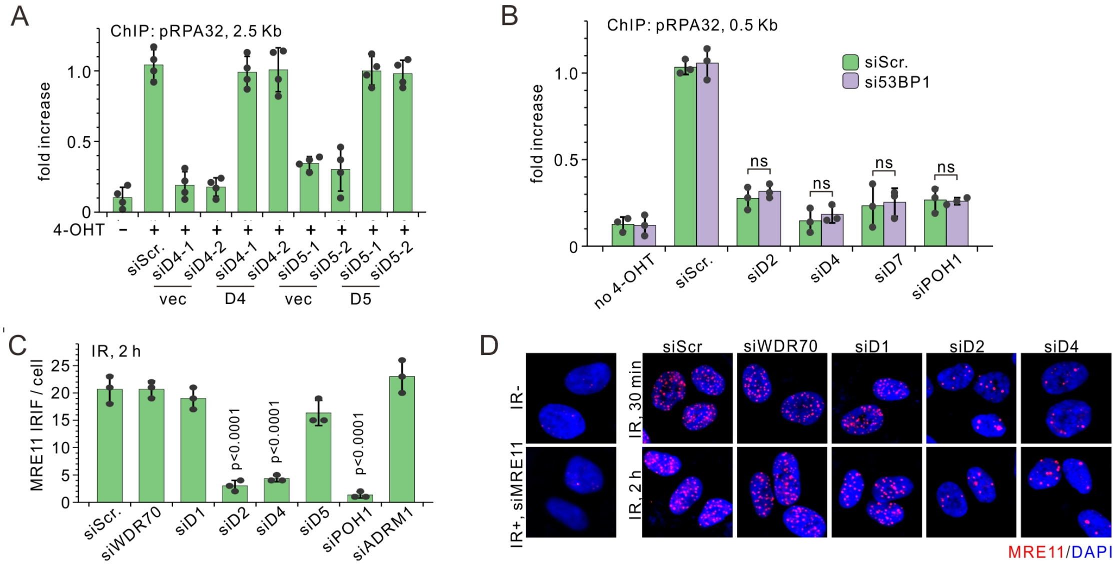

Supplemental Figure 3. The modular functions of CDW19S.

(A) DIvA cells transfected with two different sets of siRNA for *PSMD4*$^{\text{Rpn10}}$ and *PSMD5*$^{\text{Hsm3}}$. The DSB loading of pRPA32 was quantified at 2.5 Kb from an *Asi*SI site with concomitant expression of siRNA resistant plasmids or not.

(B) pRPA32 loading at 0.5 Kb from an *Asi*SI-dependednt DSB upon *PSMD2*$^{\text{Rpn1}}$, *PSMD4*$^{\text{Rpn10}}$, *PSMD7*$^{\text{Rpn8}}$ and *POH1*$^{\text{Rpn11}}$ depletion in the presence or absence of concomitant si*53BP1*.

(C,D) Quantification (C) and representative images (D) for MRE11 IRIF at the indicated time points after siRNA treatment for selected CDW19S genes.

For all graphs: n = 3 biological repeats. Error bars = s.d. p values by t-test shown for indicated groups. ns: no significant difference.

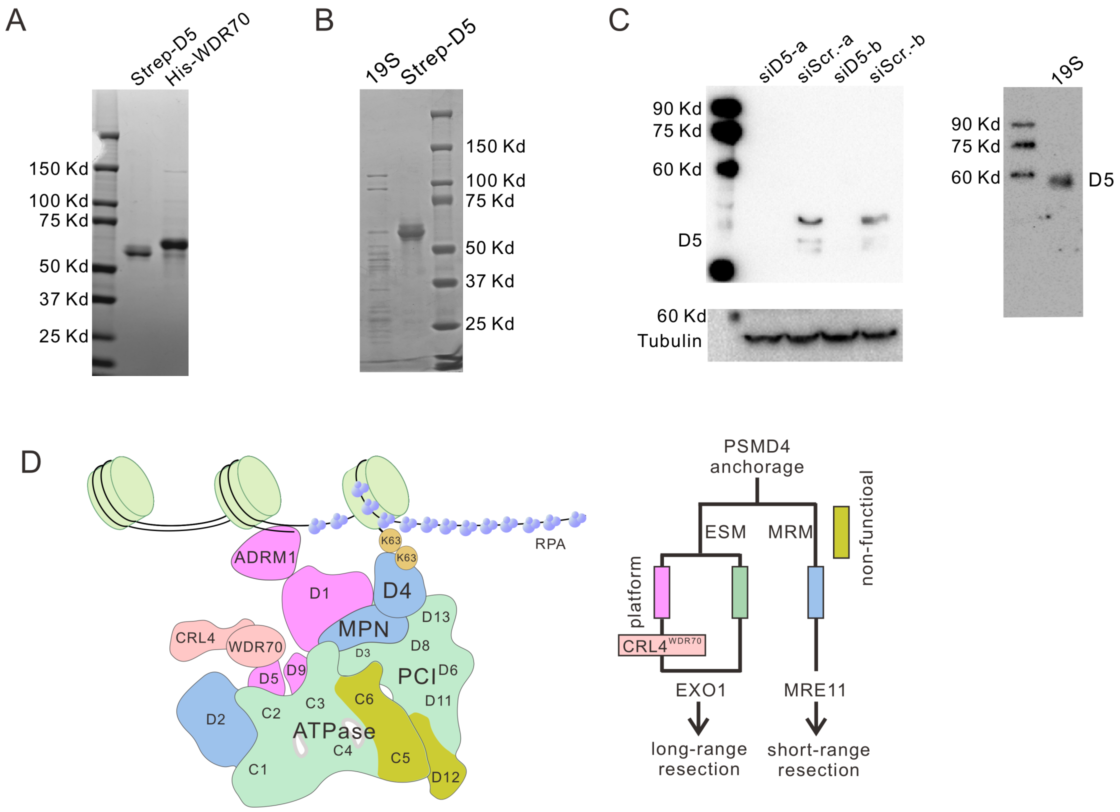

Supplemental Figure 4. Characterising the interaction between CRL4^{WDR70} and RP subunits.

(A,B) Coomassie staining showing purified recombinant WDR70 (0.5 µg) from insect cells and PSMD5^{Hsm3} (0.5 µg) from *E. coli* with indicated tags (A), plus purified commercial 19S proteasome (1 µg, R&D, E-367, B).

(C) Left: antibody specificity of α-PSMD5^{Hsm3} shown by immunoblotting. 293T cells were pre-treated with si*PSMD5*^{Hsm3} or not in two individual experiments. Right: immunoreaction detected by α-PSMD5^{Hsm3} in purified19S particles.

(D) Illustration for functional modules in CDW19S. The architecture of 19S is based on Lauren Budenholzer's description (Budenholzer et al., 2017).

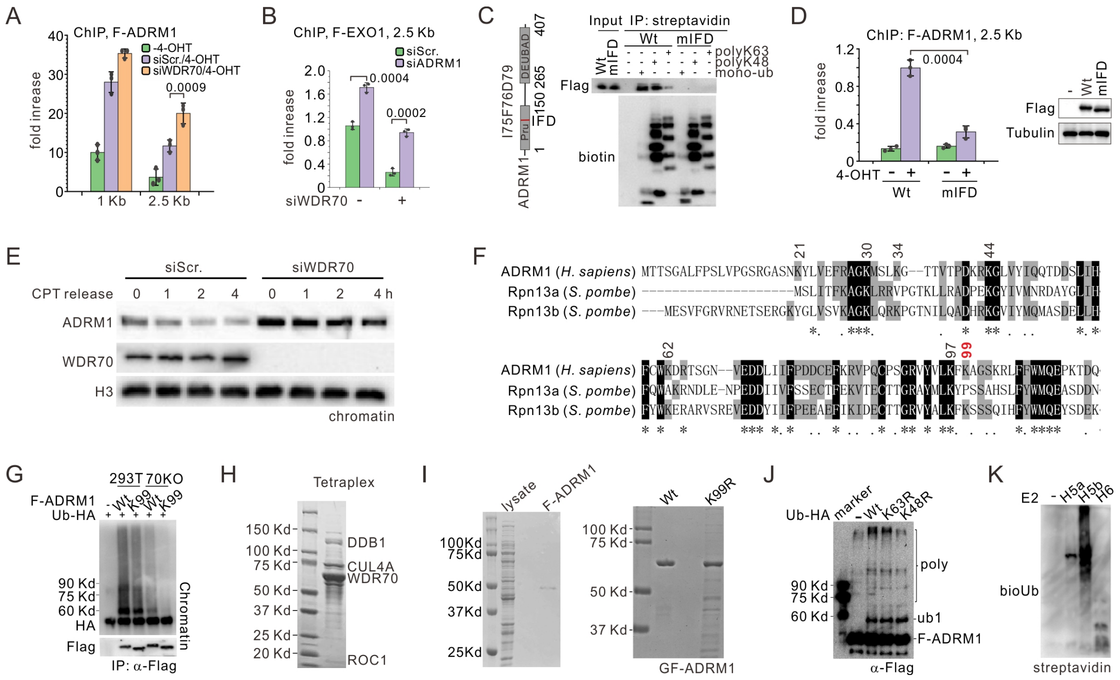

Supplemental Figure 5. ADRM1^{Rpn13} as the direct ubiquitin target of CRL4^{WDR70}.

(A,B) ChIP for ADRM1^{Rpn13} (A) and EXO1 (B) at the indicated distances from an *Asi*SI-dependent DSB in the absence of *WDR70*, *ADRM1*^{Rpn13}, or both.

(C) Schematic for ADRM1^{Rpn13} protein functional domains (left) and ubiquitin binding assay (right) for ADRM1^{Rpn13} with biotinylated ubiquitin monomer, K48 and K63 chains (0.5 μg of each) that immobilized on streptavidin beads. Flag-tagged ADRM1^{Rpn13} (wild type and mIFD) was purified from 293T cells and probed by α-Flag.

(D) Left: ChIP assay at 2.5 Kb from an *Asi*SI-dependent DSB for Flag-tagged ADRM1^{Rpn13}. Right: expression of wild type and *mIFD* monitored by α-Flag.

(E) Representative immunoblotting measuring levels of ADRM1^{Rpn13} following CPT treatment as in Figure 5B.

(F) Alignment of human and *S. pombe* ADRM1^{Rpn13} homologues, positions of conserved lysines are labelled. Note that only the human and yeast Rpn13b contain the K99 residue.

(G) Ub-pulldown assay in 293T and *WDR70*-depleted cells upon expressing Wt and K99-only Flag-ADRM1^{Rpn13} protein.

(H,I) Coomassie staining for purified CRL4^{WDR70} tetramer from sf9 cells (H) and Flag- or GST-Flag-ADRM1^{Rpn13} from *E. coli* (I).

(J,K) Ubiquitin reconstitution using indicated ubiquitin mutants (J) and E2 enzymes (K). Ubiquitinated ADRM1^{Rpn13} was monitored by streptavidin for biotinated ubiquitin in (K). For all graphs: n = 3 biological repeats. Error bars = s.d. *p* values by *t*-test shown for indicated groups. ns: no significant difference.

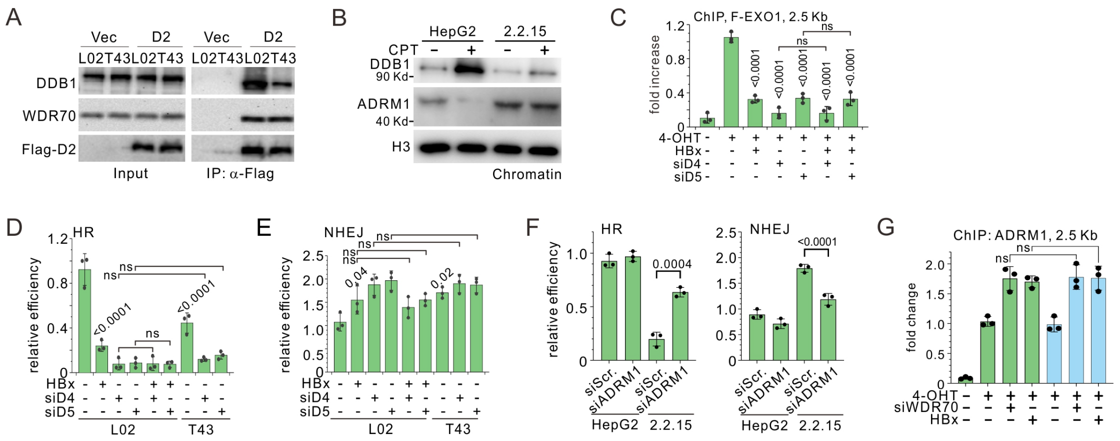

Supplemental Figure 6. HBx interferes with CDW19S organization.

(A) co-Immunoprecipitation of endogenous DDB1 and WDR70 by Flag-tagged PSMD2^{Rpn1} from L02 or T43 cell extracts.

(B) As in Figure 6B, chromatin fractions of DDB1, ADRM1^{Rpn13} and H3 were monitored by immunoblotting. Proteins were obtained from the same set of biological samples and resolved on different concentration of PAGE gels.

(C) ChIP assay for Flag-EXO1 DSB loading in the presence of indicated treatments. Values normalized to control EXO1 with 4-OHT induction.

(D,E) Repair efficacies for HR (D) and NHEJ (E) measured by I-SceI DSB system in L02 and T43 cells with indicated treatments. Statistical significance is shown relative to control cells or as indicated.

(F) Repair evaluation in HepG2/HepG2.2.15 cells with transfecting si*ADRM1*^{Rpn13} or not.

(G) ChIP for ADRM1^{Rpn13} in DIvA cells pre-transfected with si*WDR70* or *HBx* plasmid. Green: si*Scramble*, Cyan: si*53BP1*. *p* values in this figure are shown by two-way ANOVA test for multiple-groups comparison.

A B C

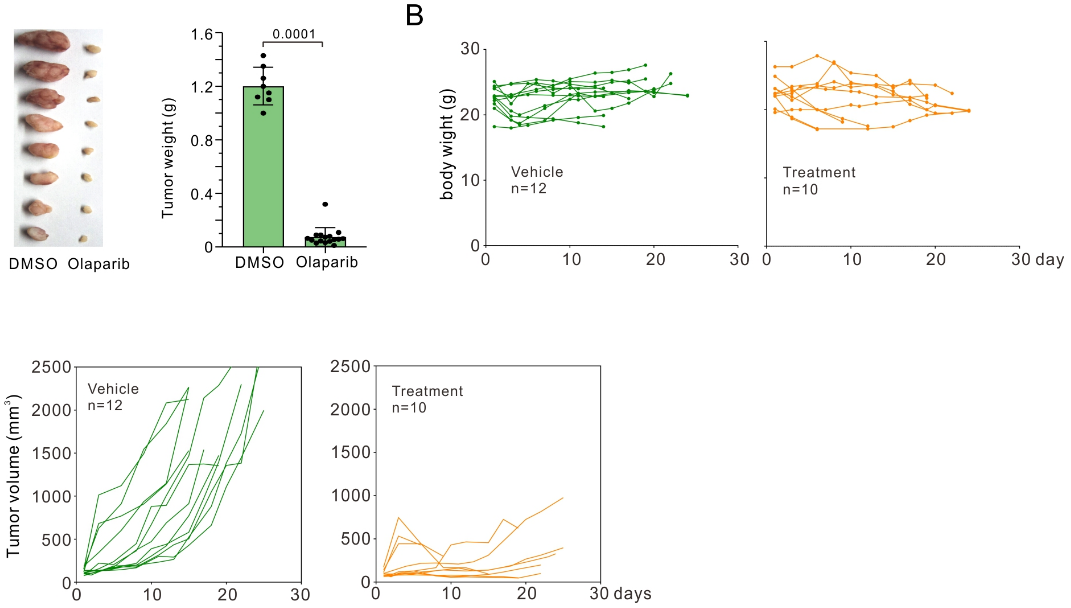

Supplemental Figure 7. Targeting HRD curbs disease progression of HBV-positive tumours.

(A) Dissected tumour (left) and average weight (right) of T43 xenografts obtained at endpoints of experiments in Figure 7A.

(B,C) Same experiments as in Figure 7D showing longitudinal individual-body-weight curves for tumour-burdened mice (B), or individual tumour-growth curves (C) of HBVHCC xenograft sublines in vehicle (green) or Olaparib + Cisplatin (O/C: orange) treated groups.

Supplemental Figure 8. The cell line identity of L02 cells.

(A) Resembling hepatocytes including HepaG2, L02 cells secret human albumin that was monitored by ELISA (Sangon Biotech, D711383), which specifically detects human but not bovine albumin. As albumin is only synthesized by hepatocytes, L02 is of liver origin.

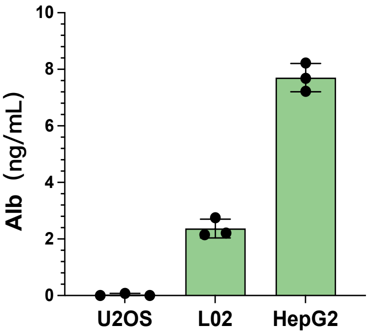

(B) The STR profile of L02 line used in this study. It shows anastomotic genotype with L02 STR in Cellosaurus database, but not with the Hela profile. This excludes the possibility that the current L02 cells was contaminated by the Hela line.

Loci	L02 in this study			L-02		Hela	
	Identified by BIOWING，China			Cellosaurus L-02 (CVCL_6926)		Cellosaurus HeLa (CVCL_0030)	
	Allele1	Allele2	Allele3	Allele1	Allele2	Allele1	Allele2
D5S818	11	12		11	12	11	12
D13S317	13.3	13.3		13.3	13.3	12	13.3
D7S820	12	12		12	12	8	12
D16S539	9	10		9	10	9	10
VWA	16	16		16	18	16	18
TH01	7	7		7	7	7	7
AMEL	X	X		X	X	X	X
TPOX	12	12		12	12	8	12
CSF1PO	10	10		10	10	9	10
D12S391	20	20				20	25
FGA	18	21		18	21	18	21
D2S1338	17	17				17	17
D21S11	27	28		27	28	27	28

D18S51	16	16		16	16	16	16
D8S1179	12	12		12	12	12	13
D3S1358	15	18		15	18	15	18
D6S1043	18	18					
PENTAE	7	17		7	17	7	17
D19S433	13	13				13	14
PENTAD	8	15		8	15	8	15
D1S1656	12	15				12	15

Supplemental Table 1. Peptide identification by mass spectrometry for spWdr70-interacting proteins

**Gene names**	**Coverage** (%)	**MW** (kDa)	**Description**
wdr70/SPAC343.17c	90.55	63.6	Uncharacterized WD repeat-containing protein
rpn2/SPBC17D11.07c	57.68	107.2	19S proteasome regulatory subunit
rpt6/SPBC23G7.12c	66.61	45.0	19S proteasome base subcomplex ATPase subunit
rpt3/SPCC576.10c	39.93	43.5	19S proteasome base subcomplex ATPase subunit
rpt4/SPCC1682.16	55.83	43.6	19S proteasome base subcomplex ATPase subunit
rpn7/SPBC582.07c	57.03	46.7	19S proteasome regulatory subunit
rpn5/SPAC1420.03	34.57	51.6	19S proteasome regulatory subunit
rpn6/SPAC23G3.11	23.71	47.3	19S proteasome regulatory subunit
rpt5/SPAC3A11.12c	37.38	48.8	19S proteasome base subcomplex ATPase subunit
rpt1/SPBC16C6.07c	35.84	48.9	19S proteasome base subcomplex ATPase subunit
rpt2/SPBC4.07c	10.04	50.0	19S proteasome base subcomplex ATPase subunit
rpn3/SPBC119.01	47.06	57.3	19S proteasome regulatory subunit
rpn1/SPBP19A11.03c	48.33	97.9	19S proteasome regulatory subunit
pcu4/ SPAC3A11.08	1.5	85.3	Cullin-4
rpn9/SPAC607.05	4.99	43.4	19S proteasome regulatory subunit
rpn1302/SPCC16A11.16c	15.46	43.9	19S proteasome regulatory subunit Rpn13b
hht1 / SPAC1834.04	5.15	15.3	histone H3 h3.1
htb1 / SPCC622.09	9.52	13.8	histone H2B

Supplemental Table 2. Plasmids used in this study

Plasmids	Source
pcDNA3-HA-HBx	Previous study
pCMV-Flag-WDR70	Previous study
pLVX-puro-HA-HBx	Previous study
pLVX-shWDR70	Purchased from Genechem
pLVX-sh53BP1	Purchased from Genechem
pCMV-NHEJ	Gift from Prof. Jun Chen, Zhejiang University
pCMV-HR	Gift from Prof. Jun Chen, Zhejiang University
pCMV-SSA	Gift from Prof. Jun Chen, Zhejiang University
pMC1-1-p84-g1	gRNA vector for *PPP1R12C/p84* locus, Purchased from Viewsolid biotech
pMC1-1-p84-g0	gRNA vector (empty), Purchased from Viewsolid biotech
pLVX-G-BRCA1	This study
pLVX-Flag-PSMD1	This study
pLVX-Flag-PSMD2	This study
pLVX-Flag-PSMD3	This study
pLVX-Flag-PSMD4	This study
pLVX-Flag-PSMD5	This study
pLVX-Flag-PSMD6	This study
pLVX-Flag-PSMD7	This study
pLVX-Flag-PSMD8	This study
pLVX-Flag-PSMD9	This study
pLVX-Flag-PSMD10	This study
pLVX-Flag-PSMD11	This study
pLVX-Flag-PSMD12	This study
pLVX-Flag-PSMD13	This study
pLVX-Flag-POH1	This study
pLVX-Flag-PSMC1	This study
pLVX-Flag-PSMC2	This study
pLVX-Flag-PSMC3	This study
pLVX-Flag-PSMC4	This study
pLVX-Flag-PSMC5	This study
pLVX-Flag-PSMC6	This study
pLVX-Flag-PSMD4-dUIM	This study
pLVX-PSMD4	This study
pLVX-PSMD4-UIM	This study
pFastBacHTA-6His-WDR70(112-654 aa)	This study
pLVX-Flag-ADRM1	This study
pLVX-Flag-ADRM1-I75RF76RD79N	This study
pLVX-Flag-ADRM1-Pru-d	This study

pLVX-Flag-ADRM1-K99R	This study
pLVX-Flag-ADRM1-K99-only	This study
pET28a-ADRM1-Flag	This study
pET28a-ADRM1-K99RFlag	This study
pFastBacHTA-6His-DDB1	This study
pFastBacHTA-6His-ROC1(5-108 aa)	This study
pFastBacHTA-6His-CUL4A(38-759 aa)	This study
pcDNA3-4HA-ubiquitin	This study
pcDNA3-4HA-ubiquitin-K48R	This study
pcDNA3-4HA-ubiquitin-K63R	This study

Supplemental Table 3. Primers used in this study

	Primers	Application
Primer 1	ATCATGGCCGACAAGCAGAAGAACG	efficiency of DSB repair, Normalize, forward
Primer 2	CGGCGGCGGTCACGAACTCC	efficiency of DSB repair, Normalize, reverse
Primer 3	TGACCACCCTGACCTACG	efficiency of DSB repair, HR and SSA repair, forward
Primer 4	CACCTTGATGCCGTTCTTCTGC	efficiency of DSB repair, repair, reverse
Primer 5	TCGGAGCAAGCTTGATTTAGGTGA	efficiency of DSB repair, NHEJ repair, forward
Primer 6	CTAACTTTGGCTCTTCACCT	Amplicon at 0.5 Kb, forward
Primer 7	GATGGAGAAAGAGAAAGGGA	Amplicon at 0.5 Kb, reverse
Primer 8	TCGCCAGTGCTTTTTCTTTT	Amplicon at 3.5 Kb, forward
Primer 9	GTTGGGGGATGATGAAAATG	Amplicon at 3.5 Kb, reverse
Primer 10	GGCTGGAGTGCAATGGCATG	Amplicon at 5 Kb, forward
Primer 11	AGACCAGCCTGGTCAACAT	Amplicon at 5 Kb, reverse
Primer 12	TCCTGCAGAAATTGCTCATAAC	Amplicon at 6 Kb, forward
Primer 13	ACGGCTGAGGGTCTTTCCAGT	Amplicon at 6 Kb, reverse
Primer 14	GCCAACCTGACCAACATGG	Amplicon at 7 Kb, forward
Primer 15	AGTGGCACGATCTTGGCTCA	Amplicon at 7 Kb, reverse
Primer 16	GACCAGCCTGGCCAACATG	Amplicon at 8 Kb, forward
Primer 17	CTGTTGCCCAGGCTGGAGTG	Amplicon at 8 Kb, forward
Primer 18	ATGGCTCATGCCTGTAATCC	Amplicon at 10 Kb/ Total DNA, forward
Primer 19	CAGCCTCCCAAGTAGCTGAG	Amplicon at 10 Kb/ Total DNA, reverse

Primer 20	CCTCCCAGAGAACAAACAGC	Amplicon at 50 Kb/ Input, forward
Primer 21	GGTTCGGGTAGGTTTTTCCT	Amplicon at 50 Kb/ Input, reverse
Primer 22	GCTCAGCTAGTCTTCTTCCTC	Amplicon for Uncut, forward
Primer 23	CTTAGAGGTTCTGGCAAGGAG	Amplicon for Uncut, reverse
Primer 24	GACGACGATAAGGAATTCATGGATTTATCTGCTCTTCG CGTTG	BRCA1 to pLVX-G, forward
Primer 25	TAGTCTCGAGGAATTCTCAGTAGTGGCTGTGGGGGA TCTGGGG	BRCA1 to pLVX-G, reverse
Primer 26	CCTGGATATGAGTTTGATCAGC	Amplicon at 0.5 Kb, DIvA system, forward
Primer 27	CTCTCCTTTCGCTGACACTG	Amplicon at 0.5 Kb, DIvA system, reverse
Primer 28	AGGAATTGACTGCGGTGTTC	Amplicon at 1 Kb, DIvA system, forward
Primer 29	GGGGAGGAGGAAAGGTGTAG	Amplicon at 1 Kb, DIvA system, reverse
Primer 30	GCCATAACAGAGGGTGGAAA	Amplicon at 2.5 Kb, DIvA system, forward
Primer 31	AACTTTAGGATGGGGCTGCT	Amplicon at 2.5 Kb, DIvA system, reverse
Primer 32	CAACATCCCTGATGACTACAGAC	Amplicon at 5 Kb, DIvA system, forward
Primer 33	GGCAATAATGTTGCCTGCAA	Amplicon at 5 Kb, DIvA system, reverse
Primer 34	GACAAACGACCCCGTGAACTAC	Expression for *PSMD1*, forward
Primer 35	CAAGGACGCCAACCACAGAAG	Expression for *PSMD1*, reverse
Primer 36	CTCATCTCTGTTTCAAATCCAC	Expression for *PSMD2*, forward
Primer 37	GGCATGATATTGAGCTAACTG	Expression for *PSMD2*, reverse
Primer 38	GGGAGAAGTTTCAAGCAGATG	Expression for *PSMD3*, forward
Primer 39	TCTCGGGTGGAATAGATGTC	Expression for *PSMD3*, reverse
Primer 40	GGATTGCTACGACTGGGACTG	Expression for *PSMD4*, forward
Primer 41	CCTGCATCACGTCGTAATCATC	Expression for *PSMD4*, reverse

Primer 42	GCTTGCTTATGAGAATAGGAC	Expression for *PSMD5*, forward
Primer 43	GGCAGCACAGTGTAGTTCAGG	Expression for *PSMD5*, reverse
Primer 44	CATGCATACAGTCAGCTGCTGG	Expression for *PSMD6*, forward
Primer 45	GTTCTTGCTATCAGGTCTGTTG	Expression for *PSMD6*, reverse
Primer 46	CCACCAGATCATCTACCAGCTGCA	Expression for *PSMD7*, forward
Primer 47	GCATCCCGGTTGGCAATCTTG	Expression for *PSMD7*, reverse
Primer 48	CCTTCTTCATTGACATCCTGCTCG	Expression for *PSMD8*, forward
Primer 49	GTTCTGTGGAGGGAATGGTGGTG	Expression for *PSMD8*, reverse
Primer 50	TCTGCAAGTGGATGATGAG	Expression for *PSMD9*, forward
Primer 51	CGTGTTGGAACAAGTCTAAG	Expression for *PSMD9*, reverse
Primer 52	GCTAATCCAGATGCTAAGGACC	Expression for *PSMD10*, forward
Primer 53	GTAAATACTTGCTCCTTGGGAC	Expression for *PSMD10*, reverse
Primer 54	CCAGAGTACAGATTGAACAC	Expression for *PSMD11*, forward
Primer 55	GTTTCCAGAGCAGCTTCGTAAG	Expression for *PSMD11*, reverse
Primer 56	CTTGAGAGTCCTGCAACGGATG	Expression for *PSMD12*, forward
Primer 57	CCTGCTAATCTGTCTACTTTAGC	Expression for *PSMD12*, reverse
Primer 58	GTTGTGCCTCATGGAGATGACT	Expression for *PSMD13*, forward
Primer 59	GTCATGTGGACTCGTTTGTC	Expression for *PSMD13*, reverse
Primer 60	GACACTTCAGGACTACAGTGAAC	Expression for *PSMD14*, forward
Primer 61	GAGGTCATAAGTACATCCACATG	Expression for *PSMD14*, reverse
Primer 62	CTTTGGATCCAGCACTTATCAG	Expression for *C1*, forward
Primer 63	CATCAGACCAGCTTCTGTACAG	Expression for *C1*, reverse

Primer 64	CAGACCTGATACTTTGGATCC	Expression for *C2*, forward
Primer 65	CTCTGATGGCAAACATACCAG	Expression for *C2*, reverse
Primer 66	CTTTGACAGTGAGAAGGCTGG	Expression for *C3*, forward
Primer 67	GTAGTTCACGTCAGGACTGAC	Expression for *C3*, reverse
Primer 68	CTTCATAGACGAGATTGATGCC	Expression for *C4*, forward
Primer 69	CATCTTGCTAGTGATAGTGGAG	Expression for *C4*, reverse
Primer 70	GATTGATATCCTGGACTCGGCAC	Expression for *C5*, forward
Primer 71	CTCAAAGTCCTCCTGAGTGACATG	Expression for *C5*, reverse
Primer 72	GATCATGGCTACAAACAGACCAG	Expression for *C6*, forward
Primer 73	CATGATCAGCACGAATTGCGAAC	Expression for *C6*, reverse
Primer 74	ACACTGTTACAAGAGTCCGACCACTG	Amplicon at 1 Kb, resection assay, forward
Primer 75	GCATATGCTGCCCAACTTTGGCTAATTC	Amplicon at 1 Kb, resection assay, reverse
Primer 76	ACCCTACAGTAGTACTAAGAAGGAC	Amplicon at 3.3 Kb, resection assay, forward
Primer 77	ATCTGGCTAGGAAACATTAGTTTAATATTC	Amplicon at 3.3 Kb, resection assay, reverse
Primer 78	GATTGGCTGTGGAGTGTGACTCA	Amplicon for total DNA, resection assay, forward
Primer 79	ATCCTCCATCTTGTCCCCTT	Amplicon for total DNA, resection assay, reverse

Supplemental Table 4. Antibodies used in this study

Antibodies	Source	dilutions
Rabbit anti-phosphor-Serine 33, RPA32	NOVUS, NB100-544	1:3000
Mouse anti-RPA32	Abcam, ab16855	1:500
Mouse anti-α-Tubulin	Sigma, T6074	1:5000
Rabbit anti-RAD51	Proteintech, 14961-1-AP	1:500
Rabbit anti-53BP1	Bethyl, A300-272A	1:1000
HRP-conjugated anti-mouse IgG	DAKO, P0260	1:2000
HRP-conjugated anti-rabbit IgG	DAKO, P0448	1:3000
FITC- conjugated anti-mouse IgG	Sigma, F0257	1:300
CY3- conjugated anti- rabbit IgG	Sigma, C2306	1:300
Rabbit anti-BRCA1	Santa Cruz, sc-6954	1:500
Mouse anti-γH2AX	Millipore, 05-636	1:500
Rabbit anti-Flag	HuaBio, custom made	1:2000
Mouse anti-PSMD5	Abnova，H00005711-M01	1:500
Rabbit anti-MRE11	CST, 4895S	1:500
Rabbit anti-WDR70	Bethyl, A301-871A	1:500
Rabbit anti-DDB1	Epitomics, 3821-1-1	1:500
Mouse anti-ubiquityl H2B	Millipore, 05-1312	1:1000
Rabbit anti-Strep	HuaBio, HA500061	1:2000
Rat anti-HA	Roche, 11867423001	1:500
Rabbit anti-GST	ZENBIO, 300195	1:2000
Rabbit anti-ADRM1	Proteintech, 11468-1-AP	1:500
Mouse anti-H3	Millipore, 05-1341	1:20000
HRP-conjugated streptavidin	Sigma, S2438	1:3000

Supplemental Table 5. siRNA used in this study

Gene	Sequence	Source
siWDR70	5'-CUGCCAGAAUGGAAGCAUA-3'	Ribobio
siDDB1	5'-CCUGUUGAUUGCCAAAAAC-3'	Ribobio
si53BP1	5'-GGCCUUUGCCUCUCAACAA-3'	Ribobio
siBRCA1	5'-GGAACCUGUCUCCACAAAG-3'	Ribobio
si*PSMD1*	5'-GAGGCAUCAUCAUUCUGAA-3'	Ribobio
si*PSMD2*	5'-GAAUGCUGGUUACGUUUGA-3'	Ribobio
si*PSMD3*	5'-GCCGCAAAGUGUUACUAUU-3'	Ribobio
si*PSMD4-1*	5'-GCACCGACAAGGCAAGAAU-3'	Ribobio
si*PSMD4-2*	5'-GGCGGAAUCAGCAGACAUU-3'	Ribobio
si*PSMD5-1*	5'-GGAUGACAGAAUCCUGGUU-3'	Ribobio
si*PSMD5-2*	5'-GCGACAAUAUCUUGCUCAA-3'	Ribobio
si*PSMD6*	5'-GAGCGAAAUUCGCGAUGCA-3'	Ribobio
si*PSMD7*	5'-UGGUCAUCAUUGAUGUGAA-3'	Ribobio
si*PSMD8*	5'-GCAUGUACGAGCAACUCAA-3'	Ribobio
si*PSMD9*	5'-CCAGCUUAGACUUGUUCCA-3'	Ribobio
si*PSMD10*	5'-AGAGUAUUCUGGCCGAUAA-3'	Ribobio
si*PSMD11*	5'-GCCUUAACUUCUGCUCGAA-3'	Ribobio
si*PSMD12*	5'-GUACUUAUGUUGAGGAAAU-3'	Ribobio
si*PSMD13*	5'-GGUUCACAGUCGUUUCUAU-3'	Ribobio
si*POH1-1*	5'-AUACCGUCAGAGUGAUUGA-3'	Ribobio
si*POH1-2*	5'-AAGGCCGGAGAUGGUUGUU-3'	Ribobio
si*PSMC1*	5'-GGAGACCUAUGCAGAUAUU-3'	Ribobio
si*PSMC2*	5'-GGUCAGAGCACUUACUCUA-3'	Ribobio
si*PSMC3*	5'-GGAGGAUGGUGCCAAUAUU-3'	Ribobio
si*PSMC4*	5'-GGAAGACCAUGUUGGCAAA-3'	Ribobio
si*PSMC5*	5'-GGAACGAACUAAAUGCUAA-3'	Ribobio
si*PSMC6*	5'-GUUCUAUUGUAGACAAGUA-3'	Ribobio
siRNF8	001: 5'-GGACAAUUAUGGACAACAA-3'<br>002: 5'-GGACGAGGAUUUGGUGUCA-3'<br>003: 5'-GCUAGAGAAUGAGCUCCAA-3'	Ribobio
siRNF168	001: 5'-GCAGUCAGUUAAUAGAAGA-3'<br>002: 5'-GUGGAACUGUGGACGAUAA-3'<br>003: 5'-CCUACAGCCUAGCAUUUCA-3'	Ribobio
siMRE11	001: 5'-GCCTCGAGTTATTAAGAAA-3'<br>002: 5'-GGATATTGTTCTAGCTAAT-3'<br>003: 5'-GGAAATGATACGTTTGTAA-3'	Ribobio
siRNF20	001: 5'- GCUAAACAGUGGAGAUAAU -3'<br>002: 5'- CCAAUGAAAUCAAGUCUAA -3'<br>003: 5'- GGAGAAGGAUGAUGCAAAU -3'	Ribobio
